# Does CVID exist in children? A genetic architecture and manifestation map derived from 7,525 patients

**DOI:** 10.70962/jhi.20260091

**Published:** 2026-07-23

**Authors:** Antonios Gkantaras, ESID Registry Working Party, Dalia Abd Elaziz, Sohilla Lofty M. Abdelkader, Avigaelle Abitbol, Hassan Abolhassani, Lalash Abrahim, Mohamed Abuzakouk, Pietro Andrea Accardo, Nabila Achir Moussouni, Veronica Afonso, Philipp Agyeman, Martina Ahlmann, Alessandro Aiuti, Abla Akl, Güzide Aksu, Kim Albers, Michael H. Albert, Meda Diana Alecsandru, Olga Aleinikova, Svetlana Aleshkevich, Radwa Salah Eldeen Youssif Alkady, Luis Allende, Mickaël Alligon, Zoe Allwood, Manrique de Lara Laia Alsina, Daniela Ambrosch-Barsoumian, Lisa Ameshofer, Kenza Amour, Evrim Anadol, Ariharan Ananthachagaran, Chantal Andriamanga, Julia Andris, Karin Andritschke, Federica Angelini, Tobias Ankermann, Katrin Apel, Siamak Arami, Ömür Ardeniz, Peter Arkwright, Karina Arnold, Rudolf Ascherl, Najla Assam, Nurit Assia-Batzir, Faranaz Atschekzei, Sybille Aumann, Volker Aumann, Magnus Aurivillius, Bernd Ausserer, Tadej Avcin, Sezin Aydemir, Emel Aygören-Pürsün, Cagdas AyvazDeniz, Chiara Azzari, Lo Babacar, Perrine Bach, Sophie Bachmann, Peter Bader, Shahrzad Bakhtiar, Renate Bancé, Catherine Bangs, Katerina Barfusz, Safa Baris, B. BarlanIsil, Michaela Bartsch, Lucia Augusta Baselli, Laura Batlle-Maso, Helge Baumann, Ulrich Baumann, Veronika Baumeister, Helen Baxendale, Suzanne Bazen, Beatrice Beaurain, Julien Beauté, Mounia Bechar-Makhloufi, Norbert Beck, Brigitta Becker, Christian Becker, Uta Behrends, Renata Beider, Rita Beier, Amel Belkacem, Luisa Belke, Sven Bellert, Bernd H. Belohradsky, Aouatef Ben-Bouzid, Vincent Benoît, Lilia BenSlama, Thamila Berdous-Sahed, Jan Bergils, Anna Berglöf, Peter Bergman, Katarzyna Bernat-Sitarz, Jolanta Bernatoniene, Ewa Bernatowska, Benedikt Bernbeck, Elena Bertolini, Claire Bethune, Kai Beuckmann, Malini Bhole, Anika-Kerstin Biegner, Stefan Bielack, Kirsten Bienemann, Arndt Bigl, Amélie Bigorgne, Marc Bijl, Nadine Binder, Michaela Bitzenhofer-Grüber, Geraldine Blanchard Rohner, Dagmar Blank, Claudia Blattmann, Julia Blau, Audra Blaziene, Stefan Blazina, Markéta Bloomfield, Roswitha Blume, Barbara Boardman, Sebastian Bode, Jaap-Jan Boelens, Christoph Boesecke, Delfien Bogaert, Johannes Bogner, Nadezda Bohynikova, Claire Booth, Victoria Bordon, Arndt Borkhardt, Melanie Börries, Michael Borte, Stephan Borte, Lukas Bossaller, Madeleine Bossard, Jeannet Botros, Soraya Boucherit, Jeannette Boutros, Oksana Boyarchuk, Onur Boyman, Kaan Boztug, Sara Branco Pereira da Silva, Thomas Braschler, Sophie Bravo, Robbert Bredius, Philip Bright, Carolina Brito de Azevedo Amaral, Nicholas Brodszki, Grit Brodt, Allan Brolund, Pauline Brosselin, Bastian Brummel, Sonja Brun-Schmid, Jürgen Brunner, Roswitha Bruns, Christina Buchta, Dietke Buck, Aileen Bücker, Matthew Buckland, Martina Bührlen, Stefan Burdach, Siobhan Burns, Janet Burton, Luis Caminal, Caterina Cancrini, Fabio Candotti, Clementina Canessa, Andrew J. Cant, Nathan Cantoni, Brindusa Capilna, Fabiola Caracseghi, Isabel Caragol, Javier Carbone, Maria Carrabba, Jean-Laurent Casanova, Latanya Chamberlain, Anita Chandra, Christophe Chantrain, Helen Chapel, Julie Chassot, Ronnie Chee, Matteo Chinello, Charu Chopra, Zita Chovancova, Martin Christmann, Krystyna Chrzanowska, Peter Ciznar, Karlien Claes, Carl Friedrich Classen, Martin Classen, Alexis-Virgil Cochino, Alison M. Condliffe, Selim Corbacioglu, Ana Isabel Cordeiro, Donatienne Cordier Wynar, Claire Core, Laurence Costes, Tanya Coulter, Virginie Courteille, Maria Cristina, Maria Cucuruz, Leonik Nel Dabrowska, Mandy Dähling, Mary Louise Daly, Claudia-Sabrina Daniel, Maria Giovanna Danieli, James Darroch, Graham Davies, Javier De Gracia Roldan, Narimene de Nadai, Iris de Schutter, Nathalie De Vergnes, Esther de Vries, Josine de Witte, Frans deBaets, Theo Debert, Christiane DeBoeck, Corina Defila, Judith Deimel, Diane Delaplace, Rosa Maria Dellepiane, Nelli Dellert, Laura Delliera, Anita Delor, Ulrike Demel, John Dempster, M.M. den Os, Rosmarie Dengg, Sora Asfaw Desisa, Alexandra Desta, Drahomira Detkova, Maite Dewerchin, Romina Dieli Crimi, Dagmar Dilloo, Florentia Dimitriou, Sarah Svenja Dinges, Jasmin Dinser, Nabila Dipani, Johannes Dirks, Anna-Maria Dittrich, Lylia Djermane, Yagmur Dogru, Figen Dogu Esin, Angelika Dombrowski, Julia Dominguez Escobar, Michaela Döring, Camilla Heldbjerg Drabe, Susann Drerup, Barbara Drexel, G.J.A. Driessen, Gregor Dückers, Yasmine Dudoit, Andrea Duppenthaler, Georg Ebetsberger-Dachs, Mate Barnabas Ecser, J. David Edgar, Maartje Eekman, Stephan Ehl, Rosanna Ehrat, Eva Eisl, Olov Ekwall, Rabab El Hawary, Sabine M. El-Helou, Aisha El-Marsafy, Sarina Elbe, Suzanne Elcombe, Alia Eldash, Youssif Alkady Radwa Salah Eldeen, P.M. Ellerbroek, Roland Elling, Jane Elliott, Aisha Elmarsafy, Angelika Engelhardt, Diana Ernst, Fügen Ersoy, Stefanie Esper, Isabel Esteves, Amos Etzioni, Andrew Exley, Martin Faber, Giovanna Fabio, Gaby Fahrni, Laura Eva Faletti, Claire-Michele Farber, João Farela Neves, Emilia Faria, Henriette Farkas, Evangelia Farmaki, Maria Faßhauer, Anders Fasth, Gerd Fätkenheuer, Stefanie Faustmann, Gisela Fecker, Conleth Feighery, Cornelia Feiterna-Sperling, Perez Eduardo Fernandez-Cruz, Cordeiro Ferreira Concalo, Alice Ferster, Tobias Feuchtinger, Oliver Feyen, Daniela Finke, Alain Fischer, Sigrid Fitter, Stefan Flaschberger, Lucia Fleckenstein, Dirk Föll, Adriano Fontana, Concetta Forino, Elisabeth Förster-Waldl, Nora Franchet, Dagmar Freitag, Urs P. Frey, Hannah Margarete Frick, Elisabeth Friedel, Wilhelm Friedrich, Barbara Frisch, Lukas Frischknecht, Stephanie Fritzemeyer, Alenka Gagro, Manfred Gahr, Nermeen Mouftah Galal, Eleonora Gambineri, Agnes Gamper, Franziska Gams, Astrid Ganzow, Nicolas Garcelon, Tomaz Garcez, Marina GarciaPrat, Ann Gardulf, Janine Garibay, Birgit Garwer, Jonathan Gathmann, Benjamin Gathmann, Corinna Gebauer, Linda Geberzahn, Tilman Geikowski, Ulf Geisen, Christiane Gemander, Andrew R. Gennery, Marie Gerisch, Michael Gernert, Katrin Gerrer, Stev Gerschmann, Sujal Ghosh, Carolin Giannini, Juana Gil Herrera, Noemi Gimenez Sanz, Matthias Girndt, Ramona Girrbach, Hermann Girschick, Antonios Gkantaras, Ioannis Gkougkourelas, Dominika Gładysz, Susanne Gnatowski, Elisabeth Gnodtke, Vera Goda, Sarah Goddard, Daniela Goebel, Jean-Christophe Goffard, Sigune Goldacker, Eva Maria Gollowitsch, Manuella Gomes, Mark Gompels, Míriam González, Luis Ignacio Gonzalez Granado, Pavels Gordins, Katharina Gößling, Lisa Göschl, Lucy Gossens, Ewelina Gowin, Lea Graafen, Leo Graca, Brigita Gradauskiene-Sitkauskiene, Dagmar Graf, Norbert Graf, Elliot Grange, H. Anne Grashoff, Johann Greil, Sofia Grigoriadou, Bodo Grimbacher, Helen Gronlund, Ute Groß-Wieltsch, Teresa Guerra, Kissy Guevara-Hoyer, Mor Seny Gueye, Seny GueyeMor, Noemie Guibert, Birgit Gülnur, Tayfun Güngör, Marina Guseva, David Guzman, Marcel Haag, Gabriele Haase, Henriette Haenicke, Filomeen Haerynck, Ines Hafsa, David Hagin, Emine Haliti, Michael Hallek, Lennart Hammarstroem, Gonca Hancioglu, Rupert Handgretinger, Leif G. Hanitsch, Susanne Hansen, Tetyana Hariyan, Thomas Harrer, Pia Hassunah, Maria Hatzistilianou, Fabian Hauck, Thomas Hauser, Margje H. Haverkamp, Grant Hayman, Paul Heath, Christian Hedrich, Maximilian Heeg, Michael Heike, Martin Heimbrodt, Sabine Heine, Ulrich Heininger, Christian Heinrich, Valerie Heinz, Andreas Heitger, Matthew Helbert, Arthur Helbling, Camilla Heldbjerg Drabe, Antje Hellige, Julya Hempel, Karen Henderson, Jörg Henes, Philipp Henneke, Christian Hennig, Karin Henrichs, Martin Herbst, Walter Hermann, Manuel Hernandez, Anja Hernández, Edyta Heropolitanska-Pliszka, Richard Herriot, Friedrich Herrmann, Archana Herwadkar, Christoph Hess, Ursula Hess, Sebastian Hesse, Sonja Higgins, Anna Hilfanova, Sophie Hilpert, Chantal Hintze, Eva Hlaváčková, Isabel Hodl, Adna Hodzic, Miriam Hoernes, Christina Hoffmann, Andreas Holbro, Christiane Höllinger, Lisa Holtsch, Ursula Holzer, Dirk Holzinger, Manfred Hönig, Andrea Hönscheid, Julia Horn, Gerd Horneff, Claire Hoyoux, Nataliya Hristova, Kai Hübel, Angela Hubner, Christian Huemer, Aarnoud Huissoon, Brigitte Hülsmann, Patrick Hundsdörfer, Hans-Iko Huppertz, Kristina Huß, Sadia Hussain, Julien Husson, Tanja Hüttner-Foehlisch, Hanna Ijspeert, Aydan Ikinciogullari, Kökçü İlknur, Ninela Irga, Alexandra Jablonka, Karina Jahnz-Rozyk, Marcus Jakob, Donate Jakoby, Donate Jakoby-Gaide, Peter Jandus, Annette Jansson, Melanie Jaquet, Helene Jardefors, Edyta Jargulinska, Barbara Jauk, Milos Jesenak, Katharina Jilka, Stephen Jolles, Alison Jones, Regina Jones, Birgit Jonkman-Berk, Göran Jönsson, Hilary J. Joyce, Pricillia Juliana, Michael Kabesch, Leo Kager, Christian Kahlert, Petra Kaiser-Labusch, Ioannis Kakkas, Dirk Kamitz, Maria Kanariou, Lothar Kanz, Elif Karakoc-Aydiner, Boris Karanovic, Mutlu Kartal-Kaess, Elisabeth Käser, Terese L. Katzenstein, Hana Kayserova, Peter Kelleher, Tessa Kerre, Sara Sebnem Kilic, Gerhard Kindle, Martina Kirchner, Simone Kiwit, Ayca Kiykim, Jessica Klasen, Maja Klaudel-Dreszler, Ariane Klein, Christoph Klein, Andreas Klein-Franke, Ilona Kleine, Stefan Kleinert, Christian Klemann, Marion Klima, Adam Klocperk, Robin Kobbe, Dilara Fatma Kocacik Uygun, Melanie Koch, Yvonne Kochler, Naschla Kohistani, Marina Kojic, Antonio Kolios, Uwe Kölsch, Sylwia Koltan, Irina Kondratenko, Christoph Königs, Julia Konoplyannikova, Peter Kopac, Jana Kopp, Dieter Körholz, Julia Körholz, Pauline Korte, Larysa Kostyuchenko, Ina Kötter, Sven Kracker, Pavlina Králícková, Christof Kramm, Philipp Kramme, Máté Krausz, Wolfhart Kreuz, Johanna Krista, Gergely Kriván, Gabriele Kropshofer, Renate Krüger, Olga Krystufkova, Maria Ktistaki, Jörn-Sven Kühl, Alexander Kühn, Taco W. Kuijpers, Wietse Kuis, Silke Kullmann, Andreas Kulozik, Dinakantha Kumararatne, Ria Kümmler, Andrea Kündgen, Magdalena Kurenko-Deptuch, Cosima KussPaula, Necil Kütükcüler, Cécile Lafoix-Mignot, Beate Lamers, Peter Lanbeck, Paul Landais, Sybille Landwehr-Kenzel, Vanessa Langemeyer, Thorsten Langer, Petra Lankisch, Nadia Lanz, Manrique de Lara, Eusebia Lara-Villacanas, Lisa Laubenthal, Hans-Jürgen Laws, Ronan Leahy, Jae-Yun Lee, Andrea Lehmann, Kai Lehmberg, Patricia Lehner, Hans Leibfrit, Leoni Leistner, Loic LeMignot, Petra Lesch, Simon Leutner, Manolis Liatsis, Linda Libai Véghová, Johanna Liebel, Johannes G. Liese, Kotryna Linauskiene, Richard Linde, Martina Linßner, Conrad Ferdinand Lippert, Jiri Litzman, Pilar Llobet, Babacar Lo, Tariq Lodin, Jindrich Lokaj, David Longhino, Hilary Longhurst, Susana Lopes da Silva, Catharina Lorenzen, Vassilios Lougaris, Doris Löw, Annelie Lubatschofski, Mary Lucas, Verena Lutz-Wiegers, Sabine Maaß, Maria Elena Maccari, Anna Macura-Biegun, Vanessa Maerz, Paraskevi Maggina, Nizar Mahlaoui, Hannah Mahrenholz, Sarah Maier, Mounia Makhlouf, Anne Malfroot, Laura Malinauskiene, Wilma Mannhardt-Laakmann, Ania Manson, Felicia Mantkowski, Petra Manzey, Carolina Marasco, Nufar Marcus-Mandelblit, Wolfgang Marg, Gasper Markelj, Laszlo Marodi, José Goncalo Marques, Laura Marques, Karin Marschall, Natalia Martínez, Rafael Martinez de la Ossa Saenz-Lopez, Inmaculada Martinez-Saguer, Baldassarre Martire, Antonio Marzollo, Refiloe Masekela, Katja Masjosthusmann, Tania Nicole Masmas, Nuria Matamoros, Jutta Mattern, Pearl Mau-Asam, Elizabeth McDermott, Nichole McIntosh, Karmen Mesko Meglic, Ruben Meijer, Andrea Meinhardt, Safa Meshaal, Yasmina Messaoud, Björn Meyer, Dirk Meyer-Olson, Isabelle Meyts, Romain Micol, Bozena Micoloc, Gudrun Mielke, Cinzia Milito, Tomas Milota, Siraj Misbah, Carolin Mödden, Michael Mohr, Karina Mohrmann, Mostafa Moin, Luis Molinos, Jana Möller, Sarah Möller-Nehring, Henner Morbach, Viviana Moschese, Olga Moser, Despina Moshous, Radoslaw Motkowski, Jayashree Motwani, Nermeen MouftahGalal, Carmen Müglich, Anna Mukhina, Eva Muller, Christiane Müller, Gabriele Müller, Hedi Müller, Ingo Müller, Thomas Müller, Zoe Müller, Ulf Müller-Ladner, Sarah Müller-Stöver, Esther Münstermann, Núria Murtra Garrell, Moritz Muschaweck, Miriam Mutert, Zohreh Nademi, Paru Naik, Andrea Näke, Andrea Martin Nalda, Gulnara Nasrullayeva, Nora Naumann-Bartsch, Verena Nemitz, Olaf Neth, Andreas Neubauer, Jennifer Neubert, Carla Neumann, Tim Niehues, Mara Niemuth, Alexandra Nieters, Chris Nieuwhof, Daniela Nolkemper, Sadia Noorani, Budde Adya Noorlander, Luigi D. Notarangelo, Gundula Notheis, Sanam Amelie Nowatsh, Gaelle Obenga, Suheyla Ocak, Mehmet Oker, Peter Olbrich, Anna Olipra, Heymut Omran, Prasad Oommen, Linda Opitz, Jaroslava Orosova, Solveig Oskarsdottir, Hülya Özsahin, Malgorzata Pac, Jana Pachlopnik-Schmid, Franco Pandolfi, Efimia Papadopoulou-Alataki, Theodora Papastamatiou, Anna Papatriantafillou-Schmieder, Alba Parra-Martinez, Olga Paschenko, Srdjan Pašić, Jarek Pasnik, Smita Patel, Martin Pavlík, Estela Paz Artal, Anouk Peeters, Sara Branco Pereira da Silva, Ruy Perez-Becker, Marc Perez-Guzman, Martine Pergent, Markus Perlhagen, Hans-Hartmut Peter, Marin Petrić, Michael Pfreundschuh, Pierre Philippet, Capucine Picard, Barbara Pietrucha, Leonora Pietzsch, Claudio Pignata, Monica Piquer Gibert, Kerstin Pirolt, Alessandro Plebani, Daniel E. Pleguezuelo, Katrin Pollok, Helena Pommerening, Daniela Popihn, Aleksandra Poplonek, Marina Popp, Fulvio Porta, Jan Portegys, Klara Posfay-Barbe, Judith Potjewijd, Sebastian Poulheim, Seraina Prader, Petra Prämassing-Scherzer, Martina Prelog, Johan Prevot, Arthur Price, Timothy Price, Marijke Proesmans, Johan Provot, Federica Pulvirenti, Isabella Quinti, Anna Raab, Anita Rack, Stefan Raffac, Eduardo Ramos Oviedo, Philippe Randrianomenjanahary, Anja Ranohavimparany, Maria Raptaki, Roonaka Rashidzadeh, Margit Rathwallner, Shereen Reda, Nahida Redouane, Frederico S. Regateiro, Janine Reichenbach, Bianca Reimers, Cornelia Reinhardt, Dirk Reinhardt, Anne Reinprecht, Tamara Reiß, Ismail Reisli, Eleonore Renner, Nima Rezaei, Alex Richter, Darko Richter, Nikolaus Peter Rieber, Nadja Rieckehr, Marion Riedel, Heidi Riescher, Johannes Rischewski, Nicole Ristl, Henrike Ritterbusch, Tanja Ritz, Francois Rivier, Peter Robinson, Jürgen K. Rockstroh, Joachim Roesler, Himatur Rofiah, Elizabeth Rogerson, Elisabeth Rolfes, Beate Roller, Yaryna Romanyshyn, Roberto Rondelli, Fien Roosens, Angela Rösen-Wolff, Valentina Rösler, Johannes Roth, Tobias Rothoeft, Agathe Roubertie, Gesa Rübsam, Stephan Rusch, Abraham Rutgers, Paul Ryan, Gudrun Sach, Kambis Sadeghi, Ulrike Sahrbacher, Angelika Saidi, Özden Sanal Tezcan, Silvia Sanchez-Ramon, Juan Luis Santos, Ravishankar Sargur, Ihor Savchak, Sinisa Savic, Bernhard Schaaf, Marzena Schaefer, Christina Schäfe, Eva Scharbatke, Ellen Schatorje, Uwe Schauer, Carmen Scheibenbogen, Raphael Scheible, Clemens Scheinecker, Romana Schiller, Beatrice Schilling, Freimut Schilling, Steffi Schlieben, Thilo Schmalbach, Marc Thomas Schmalzing, Pirmin Schmid, Nadine Schmidt, Reinhold Ernst Schmidt, Monika Schmitz, Dominik Schneider, Dominik T. Schneider, Reinhard Schneppenheim, Cathy Scholtes, E.H. Schölvinck, Stefan Schönberger, Stefan Schreiber, Rik Schrijvers, Andrea Schroll, Simone Schruhl, Johanna Schrum, Ralf Schubert, Catharina Schuetz, Sebastian Schuh, Ansgar Schulz, Claudia Schulz, Ilka Schulze, Hendrik Schulze-Koops, Ulf Schulze-Sturm, Eva-Maria Schumacher, Elvira Schürmann, Gesine Schürmann, Volker Schuster, Eva Schwaneck, Klaus Schwarz, Tobias Schwarz, Carolynne Schwarze-Zander, Lothar Schweigerer, Anna Sediva, Jörg Seebach, Reinhard Seger, Florian Segerer, Markus G. Seidel, Barbara Selle, Suranjith Seneviratne, Mikko Seppänen, Hatidje Shabanaj, Svetlana Sharapova, Anna Shcherbina, Benjamin Shillitoe, Anne Siepelmeyer, Kathrin Siepermann, Anna Simon, Arne Simon, Katja Simon-Klingenstein, Marija Simonovic, Merlin Simsen-Baratault, Elena Sindram, Alla Skapenko, Maiken Skarke, Malgorzata Skomska-Pawliszak, Mary Slatter, Julie Smet, C.I. Edvard Smith, Ali Sobh, Bettina Sobik, Georgios Sogkas, Michael Sohm, Xavier Solanich, Xavier Solanich-Moreno, Pere Soler Palacín, Franz Sollinger, Raz Somech, Anja Sonnenschein, Annarosa Soresina, Marijk Sornsakrin, Stavrieta Soura, Sabrina Spaccarotella, Guiseppe Spadaro, Monika Sparber-Sauer, Christof Specker, Carsten Speckmann, Lisa Speidel, Matthaios Speletas, Klaus-Daniel Stachel, Catherine Stadon, Aurélie Stanislas, Cynthia Stapornwongkul, Paulina Staus, Regina Steck, Cathal Steele, Herbert Steffin, Urs Steiner, Sandra Steinmann, Wim Stevens, Martina Stiefel, Sarah Stieger, Sophie Stiehler, Hermann Stimm, Silvia Stojanov, Matthias Stoll, Dominique Stoppa-Lyonnet, Tobias Strapatsas, Gabriele Strauß, Monika Streiter, Riet Strik-Albers, Gaby Strotmann, Héctor SuárezCasado, Jose Luis Subiza, Mikael Sundin, Birgit Süß, Fabienne Sutter, Anna Szaflarska, Monika Szemkus, Hannah Tamary, Sofia Tantou, Michael D. Tarzi, Helga Taschner, Ulf Tedgard, Carla Teixeira, R.J.M. ten Berge, Klaus Tenbrock, Sabine Tester, Ilhan Tezcan, Sonja Thalguter, Julian Thalhammer, Katharina Thoma, Moira Thomas, Fabian Thomczyk, Vojtech Thon, Adrian Thrasher, Patricia Tierney, Nadine Tietsch, Alberto Tommasini, Beate Tönnes, Hans-Peter Tony, Maria Trachana, Carmen Trapp, Lourdes Tricas, Maria Conceicao Trindade Neves, Jordis Trischler, Hugo Ubieto, Anett Uelzen, Annette Uhlmann, Jan Ullrich, Kurt Ullrich, Ekrem Ünal, Gerhard Urbanski, Simon Urschel, Aleksandra Uszynska, Angelo Vacca, N.N. Vaganov, Daniel Vagedes, Stefanie Valicevic, Florence Vallelian, Rachel T. van Beem, Charlotte van Damme, Annick van de Ven, J. Merlijn van den Berg, Michiel van der Flier, J.T. van Dissel, P.M. van Hagen, J.M. van Montfrans, Geoffrey van Ogtrop, Jacqui van Rens, Christel A.M.P. van Riel, Annet van Royen-Kerkhof, G.Th.J. van Well, Antari Vasiliki, Sirje Velbri, Jo Vencken, Alessandro Ventura, François Vermeulen, Christiane Vermylen, Dorothee Viemann, Anja Viereck, Anna Villa, Jeroen Vincke, Lisa Vinnemeier-Laubenthal, Kim Duy Vo Thi, Mirjam Voeller, Kristina Vollbach, Alla Volokha, Horst von Bernuth, Philipp von Bismarck, Rebecca Voss, Sandra Voß, Yüksel Vural, Heike Wachuga, Norbert Wagner, Per Wagström, Matthias Wahle, Volker Wahn, Nadine Wapp, Klaus Warnatz, Monika Warneke, Adilia Warris, Jan-Christian Wasmuth, Jean-Blaise Wasserfallen, Angela Wawer, Manfred Weber, Corrie M.R. Weemaes, Lisa Wege, Julius Wehrle, Stephan Weidinger, Michael Weiß, Elisabeth Weißbarth-Riedel, Antje Werner, Sara Wessel, Marco Westkemper, Monika Wicher, Lutz Wickmann, Sabine Wiegert, Monique Wiehe, Katharina Wiehler, Lydia Wiesböck, Ewa Wiesik-Szewczyk, Anthony Williams, Beate Winkler, Christel Winkler, Martina Winkler, Melanie Winkler, Uwe Wintergerst, Lukas Wisgrill, Torsten Witte, Helmut Wittkowski, Barbara Wolf, Sandra Wölke, Christina Wolschner, Beata Wolska-Kusnierz, Philip Wood, Sarita Workman, Austen Worth, Michaela Wortmann, Walter Alfred Wuillemin, Nico M. Wulffraat, Katharina Wustrau, Chloé Wyndham-Thomas, Olcay Yegin, Alisan Yildiran, Denise Yilmaz, Patrick Young, Esra Yucel, Milica Zečević, Erić Želimir, Fred Zepp, Klaus Zetzsche, Rainald Zeuner, Stefan Zielen, Martina Zimmermann, Markus G. Seidel

**Affiliations:** 1 1st Department of Pediatrics, Pediatric Immunology and Rheumatology Referral Center, “Hippokration” General Hospital of Thessaloniki, Aristotle University of Thessaloniki, Thessaloniki, Greece; 2 Styrian Children’s Cancer Research Unit for Cancer and Inborn Errors of the Blood and Immunity in Children, Division of Pediatric Hematology-Oncology, Department of Pediatrics and Adolescent Medicine, Medical University of Graz, Graz, Austria

## Abstract

Diagnosing common variable immunodeficiency (CVID) in childhood remains contentious, as monogenic inborn errors of immunity (IEIs) are increasingly recognized in CVID-like phenotypes. We analyzed 7,525 ESID Registry patients with a clinical diagnosis of CVID to investigate age-dependent genetic architecture and associated phenotypes. Among living CVID patients, monogenic defects were identified in 82 of 251 children younger than 18 years (32.7%) versus 447 of 5,225 adults (8.6%; OR: 5.18, 95% CI: 3.86–6.92). Pediatric-onset disease (<18 years) likewise demonstrated increased monogenic underpinnings (OR: 1.88, 95% CI: 1.56–2.27), strongest with onset before 4 years (OR: 2.99, 95% CI: 2.38–3.78). Monogenic “CVID” was more likely associated with immune dysregulation at presentation (OR: 1.98, 95% CI: 1.66–2.36) and negatively linked to infection-predominant manifestations (OR: 0.67, 95% CI: 0.56–0.81). Physician-entered “additional-gene” annotations suggested multigene constellations in 1.6% of patients and identified recurrently recorded variants in other IEI-associated genes, including *TCF3*, *PIK3CD*, and *KMT2D*, among unresolved cases. These findings support “pediatric CVID” as a provisional label requiring systematic genetic evaluation.

## Teaser

Gkantaras et al. analyzed 7,525 ESID Registry patients clinically diagnosed with CVID and show enrichment of monogenic inborn errors of immunity in pediatric cases. Their findings challenge pediatric CVID as a definite diagnosis and support systematic genetic evaluation in pediatric CVID-like cases, especially those with disease onset before 4 years and/or immune dysregulation at presentation.

## Graphical Abstract

**Figure:**
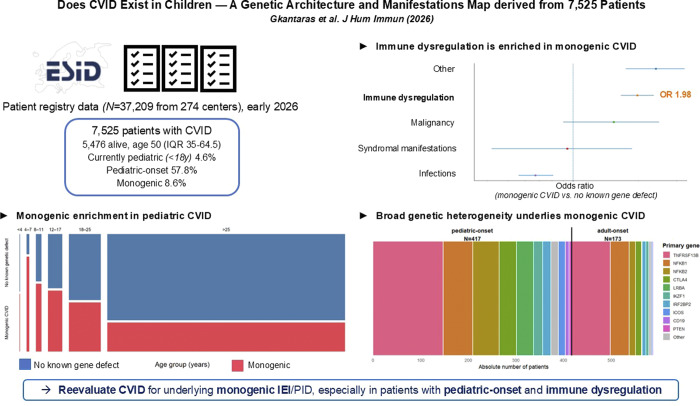

## Introduction

Common variable immunodeficiency (CVID) is an “umbrella” diagnosis encompassing a wide spectrum of predominantly antibody deficiencies with marked clinical, immunological, and genetic heterogeneity. Despite shared hallmark immunological features, including hypogammaglobulinemia and impaired antibody responses to vaccination, patients with CVID vary considerably with respect to age at disease onset, initial clinical manifestations, presence of immune dysregulation, identification of underlying pathogenic variants in immune-related genes, and long-term outcomes (1, 2). The development of clinical working definitions has been essential to navigate this heterogeneity, improving diagnostic precision and guiding clinical decision-making (3, 4, 5). According to the European Society for Immunodeficiencies (ESID) working definition, a clinical diagnosis of CVID can be established after the fourth year of life and requires the presence of CVID-like clinical features or a positive family history, marked hypogammaglobulinemia with a functional B cell deficit, and absence of profound T cell deficiency, after exclusion of secondary causes of hypogammaglobulinemia and other well-defined monogenic inborn errors of immunity (IEI) (Table S1) (4).

Simultaneously, the genetic landscape of CVID is rapidly evolving (5, 6). Historically, fewer than 10% of patients were found to have a defined monogenic cause (7). However, with the widespread application of next-generation sequencing, recent studies have demonstrated higher detection rates of monogenic (∼30%)—or occasionally oligogenic—defects in CVID-like patients, particularly in pediatric cohorts (1, 2, 5, 6, 8, 9, 10). Adding to the diagnostic complexity, identification of a pathogenic variant associated with an IEI often leads to reclassification of patients from CVID to a distinct genetically defined IEI—often within a different IEI category, according to the International Union of Immunological Societies (IUIS) classification (11, 12). This is illustrated in the ESID Registry (ESID-R), where 27% of children and 8% of adults initially diagnosed with CVID were subsequently reclassified into over 20 distinct IEI diagnoses, after applying stringent clinical criteria and incorporating genetic testing results (4). Nevertheless, within primary antibody deficiencies, the majority of patients still lack an established genetic diagnosis, and CVID represents the most frequently attributed clinical diagnosis in this genetically unresolved group (13).

Despite this evolving landscape, major gaps remain, raising a challenging question: to what extent does a CVID diagnosis in childhood represent clinically defined but genetically unresolved CVID, rather than an evolving genetically defined IEI potentially warranting targeted or definitive therapy? Diagnosis of CVID during early childhood remains contentious, as ongoing immune system maturation, transient hypogammaglobulinemia of infancy, and other pediatric-specific immune dynamics may mimic features of CVID, thereby limiting the applicability of adult-derived CVID diagnostic criteria (1). While the genetic basis of CVID is increasingly being elucidated, systematic comparisons of genetic architecture across age strata—including the frequency of monogenic defects and the potential contribution of oligogenic/polygenic inheritance—remain scarce. Moreover, although clinical differences between pediatric-onset and adult-onset CVID have been reported (14, 15, 16), large-scale or multicenter studies examining how genetic status relates to age at diagnosis, age at disease onset, and presenting clinical manifestations remain limited. A better understanding of these associations is essential for refining disease classification, improving diagnostic strategies, and identifying patient subgroups that may benefit most from early genetic testing.

Therefore, using data from 7,525 patients in the ESID-R, we investigated the age-dependent genetic architecture of CVID. Specifically, we examined whether monogenic defects are enriched among pediatric patients with CVID and whether genetic findings are associated with the presenting clinical phenotype. By addressing these questions in a large multicenter registry cohort, we aimed to inform the ongoing debate as to whether pediatric CVID represents clinically defined but genetically unresolved early-onset CVID or a biologically heterogeneous diagnostic entity enriched for various genetically defined IEI.

## Results

### Cohort characteristics

The ESID-R contained 7,525 patients with a clinical diagnosis of CVID, including 3,472 males and 4,053 females (Table 1). Overall, 646 patients (8.6%) had an identified monogenic defect. Among 5,476 patients alive at the time of analysis, 251 (4.6%) were pediatric (<18 years), whereas 3,012 (55.0%) had pediatric disease onset, and 529 (9.7%) had a monogenic defect. Across the full cohort, age-at-onset analyses were restricted to 7,234 patients with valid onset data, including 4,180 with pediatric-onset disease. Within the pediatric-onset group, disease onset occurred before 4 years in 1,574 patients, at 4–7 years in 795 patients, at 8–11 years in 440 patients, and at 12–17 years in 1,371 patients. Genetic testing was not uniformly performed across the ESID-R CVID cohort (28.5% of the cohort). Therefore, whole-cohort analyses were retained to describe registry-level burden and testing gaps, whereas analyses restricted to genetically tested patients were used as the primary denominator for interpreting age-dependent enrichment of monogenic diagnoses. This distinction is maintained throughout the Results and in the corresponding table legends.

**Table 1.** Demographic, clinical, and genetic characteristics of the analyzed CVID cohort (*N* = 7,525)

Characteristics	*N* (%) or median (IQR)
**Sex (male)**	3,472 (46.1%)
**Current age (years) (only for alive patients, ** ***N*** ** = 5,476)**	50.0 (35.0–64.5)
**Currently pediatric cases (out of alive patients, ** ***N*** ** = 5,476)**	251 (4.6%)
**Age at disease onset (years)**	14.0 (4.5–32.8)
**Pediatric-onset cases (out of patients with valid data, ** ***N*** ** = 7,234)**	4,180 (57.8%)
**Age at clinical diagnosis (years)**	32.8 (17.1–48.0)
**Diagnostic delay (years)**	6.2 (1.6–18.0)
**Initial clinical manifestations**	
Infections	6,063 (80.6%)
Immune dysregulation	1,694 (22.5%)
Syndromic manifestations	111 (1.5%)
Malignancy	160 (2.1%)
Other	317 (4.2%)
**Clinical manifestations during follow-up**	
Recurring infections (*N* = 489)	418 (85.5%)
Immune dysregulation (*N* = 443)	195 (44.0%)
Severe atopy (*N* = 430)	20 (4.7%)
Malignancy (*N* = 2,921)	347 (11.9%)
**Current treatment**	​
IgG replacement (*N* = 6,205)	5,210 (84.0%)
Immunosuppresants/biologicals (N = 2,632)	685 (26.0%)
-Corticosteroids	444 (16.9%)
-Rituximab	208 (7.9%)
Splenectomy (*N* = 4,706)	208 (4.4%)
HSCT (*N* = 7,303)	74 (1.0%)
Gene therapy (*N* = 7,284)	1 (0.01%)
**Genetically tested patients**	2,145 (28.5%)
**Age at genetic diagnosis (years)**	34.2 (19.0–50.4)
**Delay of genetic diagnosis (years)**	12.8 (5.8–23.9)
**Monogenic CVID cases**	646 (8.6%)
**Monogenic defects—affected gene**	
*TNFRSF13B*	239 (37.0%)
*NFKB1*	119 (18.4%)
*NFKB2*	74 (11.4%)
*CTLA4*	56 (8.7%)
*LRBA*	38 (5.9%)
*IKZF1*	29 (4.5%)
*IRF2BP2*	26 (4.0%)
*ICOS*	18 (2.8%)
*CD19*	11 (1.7%)
Other rare genes (<10 patients per gene)	36 (5.6%)
**Current status**	
Alive	5,476 (72.8%)
Deceased	817 (10.8%)
Lost to follow-up/unknown	1,202 (16.0%)
Discharged/complete recovery	30 (0.4%)

Denominator for percentages is shown in parentheses and corresponds to patient entries with valid and known data.

### Enrichment of monogenic diagnoses in pediatric CVID

Because genetic testing was not uniformly recorded, the primary enrichment analysis was performed among patients with documented genetic testing. In this subgroup, monogenic diagnoses were significantly more frequent among currently pediatric patients than among adults: 82 of 142 pediatric patients (57.7%) vs. 447 of 1,642 adults (27.2%) had a monogenic defect, corresponding to an odds ratio (OR) of 3.65 (95% confidence interval [CI]: 2.54–5.28; P < 0.001) (Table 2 A). The corresponding whole-cohort analysis showed the same direction of association, with monogenic diagnoses recorded in 82 of 251 pediatric patients (32.7%) vs. 447 of 5,225 adults (8.6%; OR: 5.18; 95% CI: 3.86–6.92; P < 0.001) (Table 2 B).

**Table 2.** Associations between age categories and CVID type

​	​	​	​
**(A) Association between current age and CVID type among alive and genetically tested patients (*N* = 1,784)**			
**Current-age category**	**Monogenic CVID (** ***N*** ** = 529)**	**No known genetic defect (** ***N*** ** = 1,255)**	**OR (95% CI); P value**
Pediatric (*N* = 142)	82 (57.7%)	60 (42.3%)	3.65 (95% CI: 2.54–5.28); P < 0.001
Adult (*N* = 1,642)	447 (27.2%)	1,195 (72.8%)	
**(B) Association between current-age category and CVID type among alive patients (*N* = 5,476)**			
**Current-age category**	**Monogenic CVID (** ***N*** ** = 529)**	**No known genetic defect (** ***N*** ** = 4,947)**	**OR (95% CI); P value**
Pediatric (*N* = 251)	82 (32.7%)	169 (67.3%)	5.18 (95% CI: 3.86–6.92); P < 0.001
Adult (*N* = 5,225)	447 (8.6%)	4,778 (91.4%)	
**(C) Association between age at disease onset and CVID type among genetically tested patients (*N* = 2,046)**			
**Age-at-disease-onset group**	**Monogenic CVID (** ***N*** ** = 597)**	**No known genetic defect (** ***N*** ** = 1,449)**	**OR (95% CI); P value**
Pediatric-onset (*N* = 1,303)	424 (32.5%)	879 (67.5%)	1.59 (95% CI: 1.29–97); P < 0.001
Adult-onset (*N* = 743)	173 (23.3%)	570 (76.7%)	
**(D) Association between age at disease onset and CVID type among all patients (*N* = 7,234)**			
**Age-at-disease-onset group**	**Monogenic CVID (** ***N*** ** = 597)**	**No known genetic defect (** ***N*** ** = 6,637)**	**OR (95% CI); P value**
Pediatric-onset (*N* = 4,180)	424 (10.1%)	3,756 (89.9%)	1.88 (95% CI: 1.56–2.27); P < 0.001
Adult-onset (*N* = 3,054)	173 (5.7%)	2,881 (94.3%)	

Whole-cohort analyses (sections A and C) describe registry-level burden and should not be interpreted as diagnostic yield, because genetic testing was not uniformly recorded. Analyses restricted to genetically tested patients (sections B and D) provide the primary denominator for interpreting age-dependent enrichment of monogenic diagnoses.

Using age at disease onset rather than current age, the same pattern was observed. Among genetically tested patients with available or valid age-at-onset data, 32.5% of pediatric-onset cases harbored a monogenic defect, compared with 23.3% of adult-onset cases, yielding an OR of 1.59 (95% CI: 1.29–1.97; P < 0.001) (Table 2 C). Consistently, in the whole-cohort onset-based analysis, monogenic CVID remained more frequent in pediatric-onset than adult-onset cases (1.88; 95% CI: 1.56–2.27; P < 0.001) (Table 2 D).

**Figure 1.**
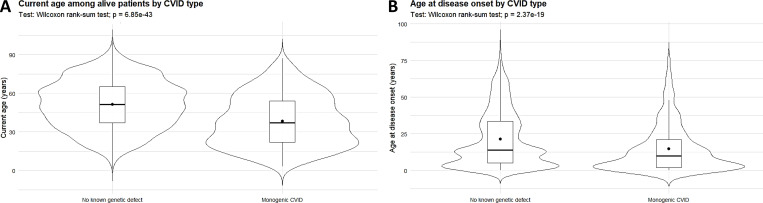
**Violin plots comparing age distributions between patients with monogenic CVID and patients with no known genetic defect. (A)** Current age among alive patients, stratified by CVID type. **(B)** Age at disease onset, stratified by CVID type. Violin plots depict the kernel density distribution of age. Embedded boxplots show the median (line) and IQR (25th–75th percentiles), with whiskers extending to 1.5 × IQR. Black dots indicate the mean. Between-group comparisons were performed using the Wilcoxon rank-sum test. IQR, interquartile range.

These patterns were further supported by continuous-age group comparisons: compared with patients without known genetic defect, those with monogenic CVID were younger among alive patients (37.0 [22.0–54.0] vs. 51.3 [37.0–65.3] years; P < 0.001) and exhibited earlier disease onset in the overall cohort (10.0 [interquartile range [IQR] 2.0–21.1] vs. 14.0 [5.0–33.6] years; P < 0.001), than their nongenetic CVID counterparts (Fig. 1).

### Age-stratified gradient in monogenic CVID diagnoses

**Figure 2.**
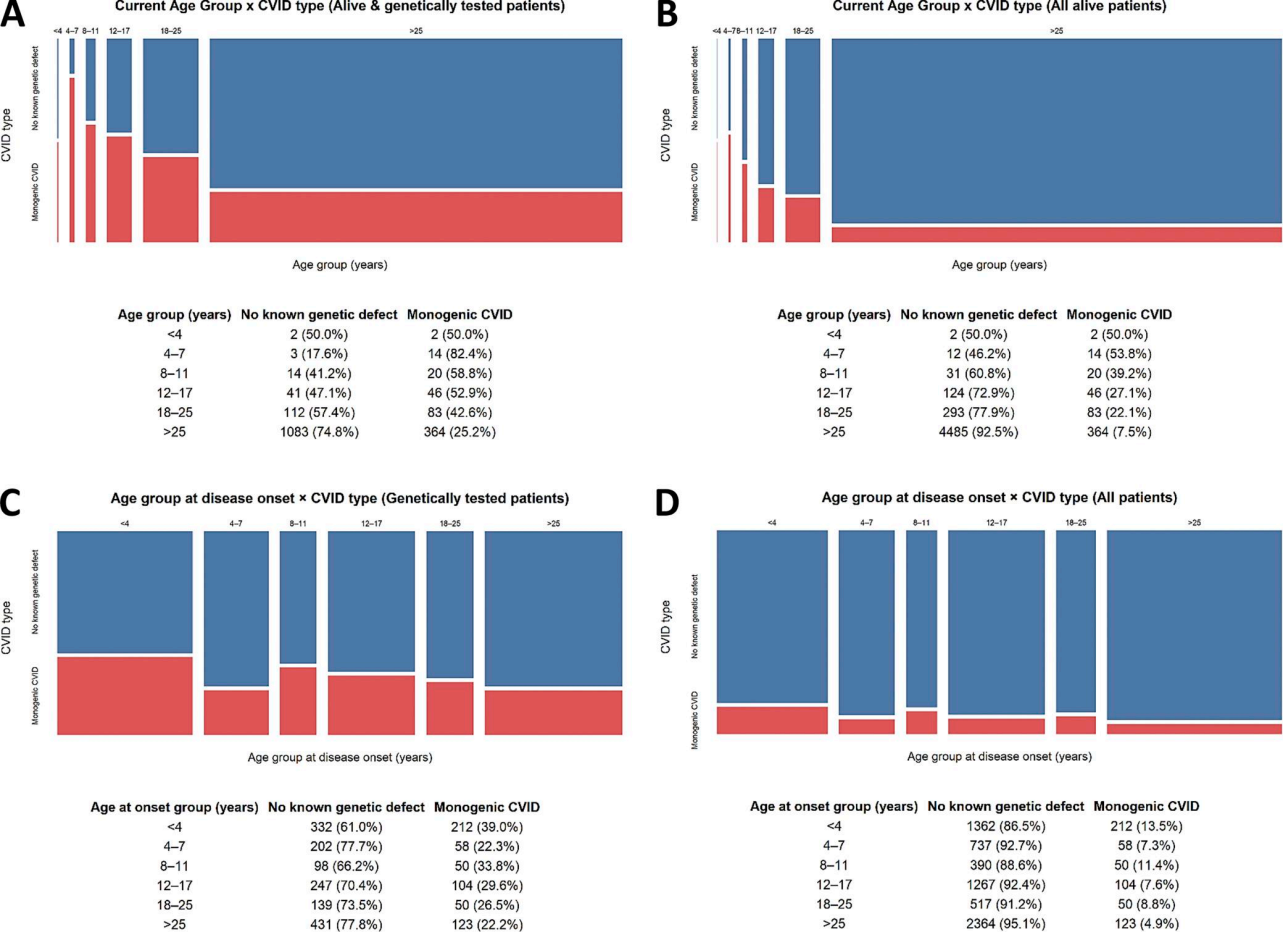
**Mosaic plots depicting the age-stratified distribution of monogenic CVID diagnoses across distinct age groups.** Mosaic plots show the proportion of patients with monogenic CVID versus no known genetic defect across predefined age strata. **(A)** Current-age group among living patients with documented genetic testing. **(B)** Current-age group among all patients alive. **(C)** Age-at-disease-onset group among genetically tested patients with available age-at-onset data. **(D)** Age-at-disease-onset group among all patients with available age-at-onset data. In each panel, columns correspond to age groups and are scaled according to the number of patients within each age stratum, while the vertical division within each column represents the relative proportion of patients with monogenic CVID and those with no known genetic defect. Red areas indicate monogenic CVID, while blue areas indicate no known genetic defect. Tables below each mosaic plot provide the corresponding absolute patient numbers and percentages within each age group. Together, the plots illustrate the age-dependent enrichment of monogenic diagnoses, particularly among younger patients and patients with pediatric disease onset, and show that this pattern persists after restriction to genetically tested individuals. Interactive HTML versions of the figure panels are available at the following links: (A) https://esid.org/html-pages/Fig2A_Interactive_Mosaic_A_CurrentAge_CVID_Filtered.html; (B) https://esid.org/html-pages/Fig2B_Interactive_Mosaic_B_CurrentAge_CVID_Alive.html; (C) https://esid.org/html-pages/Fig2C_Interactive_Mosaic_C_AgeOnset_CVID_Filtered.html; (D) https://esid.org/html-pages/Fig2D_Interactive_Mosaic_D_AgeOnset_CVID_All.html.

When current age was analyzed in predetermined finer strata, the proportion of monogenic CVID diagnoses demonstrated a clear inverse relationship with age, with the strongest enrichment of monogenic defects observed in younger age groups. Among genetically tested patients, the odds of monogenic CVID were highest among children aged 4–7 years (OR: 13.88; P < 0.001), followed by those aged 8–11 years (OR: 4.25; P < 0.001) and 12–17 years (OR: 3.34; P < 0.001), and young adults aged 18–25 years (OR: 2.20; P < 0.001), compared with patients older than 25 years (Fig. 2 A, Table 3 A). It should be highlighted that genetic testing had been performed more frequently in pediatric than adult patients (OR: 2.84; 95% CI: 2.20–3.67; P < 0.001) (Table S2 A). The corresponding whole-cohort analysis showed the same-age gradient, with the highest odds of monogenic CVID again observed in children aged 4–7 years (OR: 14.37; P < 0.001) (Fig. 2 B; Table 3 B).

**Table 3.** Associations between age groups and CVID type

​	​	​	​	​
**(A) Association between current age and CVID type among alive and genetically tested patients (*N* = 1,784)**				
**Current age (years)**	**Monogenic CVID (** ***N*** ** = 529)**	**No known genetic defect (** ***N*** ** = 1,255)**	**OR (95% CI)**	**P value**
<4	2 (50.0%)	2 (50.0%)	2.98 (0.36–24.87)	0.276 (NS)
4–7	14 (82.4%)	3 (17.6%)	13.88 (4.5–60.48)	<0.001
8–11	20 (58.8%)	14 (41.2%)	4.25 (2.14–8.67)	<0.001
12–17	46 (52.9%)	41 (47.1%)	3.34 (2.16–5.19)	<0.001
18–25	83 (42.6%)	112 (57.4%)	2.2 (1.62–3)	<0.001
>25	364 (25.2%)	1,083 (74.8%)	NA	​
**(B) Association between current-age group and CVID type among alive patients (*N* = 5,476)**				
**Current age (years)**	**Monogenic CVID (** ***N*** ** = 529)**	**No known genetic defect (** ***N*** ** = 4,947)**	**OR (95% CI)**	**P value**
<4	2 (50.0%)	2 (50.0%)	12.32 (1.47–102.94)	0.012
4–7	14 (53.8%)	12 (46.2%)	14.37 (6.59–31.85)	<0.001
8–11	20 (39.2%)	31 (60.8%)	7.95 (4.42–13.98)	<0.001
12–17	46 (27.1%)	124 (72.9%)	4.57 (3.18–6.47)	<0.001
18–25	83 (22.1%)	293 (77.9%)	3.49 (2.66–4.54)	<0.001
>25	364 (7.5%)	4,485 (92.5%)	NA	​
**(C) Association between age at disease onset and CVID type among genetically tested patients (*N* = 2,046)**				
**Age at onset (years)**	**Monogenic CVID (** ***N*** ** = 597)**	**No known genetic defect (** ***N*** ** = 1,449)**	**OR (95% CI)**	**P value**
<4	212 (39.0%)	332 (61.0%)	2.24 (1.72–2.92)	<0.001
4–7	58 (22.3%)	202 (77.7%)	1.01 (0.70–1.43)	0.973 (NS)
8–11	50 (33.8%)	98 (66.2%)	1.79 (1.20–2.65)	0.004
12–17	104 (29.6%)	247 (70.4%)	1.48 (1.09–2.00)	0.012
18–25	50 (26.5%)	139 (73.5%)	1.26 (0.86–1.84)	0.233 (NS)
>25	123 (22.2%)	431 (77.8%)	NA	​
**(D) Association between age-at-disease-onset group and CVID type among all patients (*N* = 7,234)**				
**Age at onset (years)**	**Monogenic CVID (** ***N*** ** = 597)**	**No known genetic defect (** ***N*** ** = 6,637)**	**OR (95% CI)**	**P value**
<4	212 (13.5%)	1,362 (86.5%)	2.99 (2.38–3.78)	<0.001
4–7	58 (7.3%)	737 (92.7%)	1.51 (1.09–2.08)	0.012
8–11	50 (11.4%)	390 (88.6%)	2.46 (1.73–3.46)	<0.001
12–17	104 (7.6%)	1,267 (92.4%)	1.58 (1.20–2.07)	<0.001
18–25	50 (8.8%)	517 (91.2%)	1.86 (1.31–2.60)	<0.001
>25	123 (4.9%)	2,364 (95.1%)	NA	​

Whole-cohort analyses (sections A and C) describe registry-level burden and should not be interpreted as diagnostic yield, because genetic testing was not uniformly recorded. Analyses restricted to genetically tested patients (sections B and D) provide the primary denominator for interpreting age-dependent enrichment of monogenic diagnoses. OR outcome (monogenic vs. no known genetic defect) using >25 age group as reference. NA, not applicable; NS, not significant.

When stratified by age at disease onset, genetically tested patients with disease onset before 4 years of age had nearly twofold higher odds of harboring a monogenic defect than those with onset after 25 years of age (OR: 2.24; 95% CI: 1.72–2.92; P < 0.001). More modest enrichment was observed for onset at 8–11 years and 12–17 years, whereas onset at 4–7 years and 18–25 years was not significantly enriched in the genetically tested subgroup, indicating that the association between pediatric onset and monogenic disease is driven predominantly by the earliest onset group (Fig. 2 C; Table 3 C). In addition, genetic testing was performed more frequently in pediatric-onset than adult-onset disease (OR: 1.41; 95% CI: 1.27–1.57; P < 0.001) (Table S2 B). The corresponding whole-cohort analysis showed enrichment across all onset strata below 25 years, with the largest effect again observed for onset before 4 years (OR: 2.99; 95% CI: 2.38–3.78; P < 0.001) (Fig. 2 D; Table 3 D).

Overall, these analyses support a graded, age-dependent genetic architecture, with the strongest enrichment of monogenic CVID diagnoses in childhood and preadolescence (<12 years) and in patients with the earliest disease onset (<4 years of age).

In a sensitivity analysis excluding patients with a recorded age at clinical CVID diagnosis before 4 years (*n* = 309), disease onset before 4 years remained enriched for monogenic CVID compared with onset after 25 years, both in the whole cohort (OR: 2.74; 95% CI: 2.14–3.50; P < 0.001) and among genetically tested patients (OR: 2.01; 95% CI: 1.52–2.66; P < 0.001). Thus, the enrichment observed in the earliest onset stratum was not solely driven by patients assigned a clinical CVID diagnosis before 4 years of age.

### Clinical phenotype associations with age at disease onset and genetic background

To evaluate whether the age-dependent enrichment of monogenic CVID was accompanied by phenotypic differences, we examined both initial and current clinical manifestations according to age at disease onset and genetic background. For current manifestations, analyses were restricted to patients with available follow-up data and should therefore be interpreted with caution, as a reduced-denominator, exploratory subanalysis.

**Figure 3.**
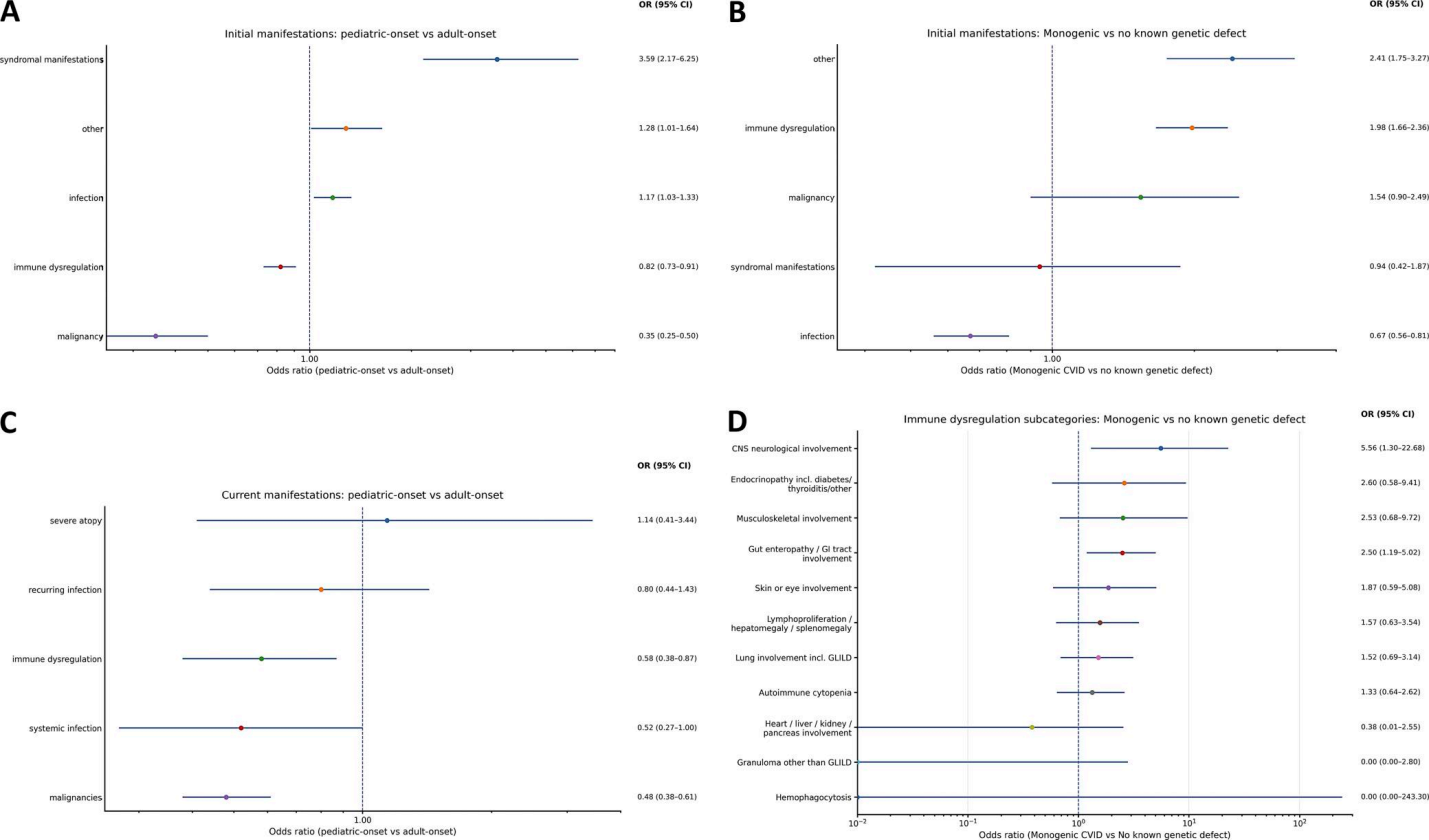
**Associations of presenting or current clinical manifestations with age or genetic background in patients with CVID.** **(** **A–D)** Forest plots illustrating associations between (A) presenting clinical phenotypes and age at disease onset; (B) presenting clinical phenotypes and genetic background; (C) current clinical manifestations and age at disease onset; and (D) specific immune dysregulation manifestations and genetic background. Current-manifestation analyses in panel C were restricted to reduced-denominator subsets, because the corresponding ESID-R fields were introduced only in December 2024. In the present extraction, current-manifestation data were available for 426 patients for immune dysregulation, 467 for recurring infections, 283 for systemic infections, 408 for severe atopy, and 2,465 for malignancy. Specific immune dysregulation subcategories shown in panel D were analyzed within the subset of patients with available immune dysregulation current-manifestation data (*N* = 426). Severe atopy, malignancy, and recurring and systemic infections were captured as ESID-R current-manifestation categories without standardized subfields in the dataset analyzed. Points indicate ORs, and horizontal bars indicate 95% CIs. The vertical dashed line denotes OR = 1, corresponding to no association between the compared variables. In panels, comparing age at disease onset (A and C), ORs >1 indicate enrichment among pediatric-onset cases. In panels comparing genetic background (B and D), ORs >1 indicate enrichment among patients with monogenic CVID. All forest plots are displayed on a logarithmic x axis. CIs, confidence intervals; CNS, central nervous system; GI, gastrointestinal; GLILD, granulomatous–lymphocytic interstitial lung disease.

At presentation, pediatric-onset CVID was associated with a distinct clinical profile. Compared with adult-onset patients, pediatric-onset CVID more frequently presented with syndromic features (OR: 3.59, 95% CI: 2.17–6.25; P_adj_ < 0.001), and, to a lesser extent, “other” manifestations (OR: 1.28, 95% CI: 1.01–1.64; P_adj_ = 0.036), and infection-predominant phenotype (OR: 1.17, 95% CI: 1.03–1.33; P_adj_ = 0.016). Syndromic features were defined as nonimmunological or multisystem manifestations suggestive of a syndromic IEI, including dysmorphism, neurodevelopmental involvement, congenital anomalies, skeletal or ectodermal features, or other organ-system manifestations recorded by the treating physician, whereas “other” manifestations included features not classifiable under the main clinical categories, such as failure to thrive, endocrine disorders, and incidental laboratory abnormalities. In contrast, immune dysregulation (OR: 0.82, 95% CI: 0.73–0.91; P_adj_ < 0.001) and especially malignancy (OR: 0.35, 95% CI: 0.25–0.50; P_adj_ < 0.001) were more common first manifestations in adult-onset cases (Table 4 A and Fig. 3 A). Among alive patients stratified by current age, pediatric CVID cases likewise demonstrated enrichment of syndromal and “other” manifestations, whereas malignancy at presentation was absent. In contrast, both infectious and immune dysregulation presenting phenotypes did not differ significantly between current pediatric and adult patients after multiple-testing correction (Table 4 B).

**Table 4.** Associations between age categories or CVID types and initial clinical manifestations

​	​	​	​	​	​
**(A) Association between age-at-disease-onset group and initial clinical manifestations among all patients (*N* = 7,234)**					
**Initial manifestation**	**Pediatric—yes (%)**	**Adult—yes (%)**	**OR (95% CI)**	**P value**	**FDR-adjusted P value**
Syndromal	92 (2.2%)	19 (0.6%)	3.59 (2.17–6.25)	<0.001	<0.001
Other	200 (4.8%)	115 (3.8%)	1.28 (1.01–1.64)	0.036	0.036
Infection	3,538 (84.6%)	2,518 (82.4%)	1.17 (1.03–1.33)	0.013	0.016
ImmDys	915 (21.9%)	779 (25.5%)	0.82 (0.73–0.91)	<0.001	<0.001
Malignancy	53 (1.3%)	107 (3.5%)	0.35 (0.25–0.50)	<0.001	<0.001
**(B) Association between current-age category and initial clinical manifestations among alive patients (*N* = 5,476)**					
**Initial manifestation**	**Pediatric—yes (%)**	**Adult—yes (%)**	**OR (95% CI)**	**P value**	**FDR-adjusted P value**
Syndromal	11 (4.4%)	45 (0.9%)	5.27 (2.43–10.52)	<0.001	<0.001
Other	25 (10.0%)	205 (3.9%)	2.71 (1.68–4.22)	<0.001	<0.001
Infection	200 (79.7%)	4,238 (81.1%)	0.91 (0.66–1.28)	0.564	0.564 (NS)
ImmDys	49 (19.5%)	1,240 (23.7%)	0.78 (0.55–1.08)	0.128	0.160 (NS)
Malignancy	0 (0.0%)	114 (2.2%)	0.0 (0–0.67)	0.001	0.017
**(C) Association between CVID types and initial clinical manifestations among all patients (*N* = 7,525)**					
**Initial manifestation**	**Monogenic CVID (*N* = 646)—yes (%)**	**No known genetic defect (*N* = 6,879)—yes (%)**	**OR (95% CI)**	**P value**	**FDR-adjusted P value**
Other	56 (8.7%)	261 (3.8%)	2.41 (1.75–3.27)	<0.001	<0.001*
ImmDys	226 (35.0%)	1,468 (21.3%)	1.98 (1.66–2.36)	<0.001	<0.001*
Malignancy	20 (3.1%)	140 (2.0%)	1.54 (0.90–2.49)	0.085	0.107 (NS)
Syndromal	9 (1.4%)	102 (1.5%)	0.94 (0.42–1.87)	1.000	1.000 (NS)
Infection	480 (74.3%)	5,583 (81.2%)	0.67 (0.56–0.81)	<0.001	<0.001*

Variables are presented in descending order of OR. ImmDys, immune dysregulation; FDR, false discovery rate; NS, not significant.

A complementary pattern emerged when presenting clinical phenotypes were investigated by genetic background. Compared with patients without a known genetic defect, monogenic CVID was more frequently associated with immune dysregulation (OR: 1.98, 95% CI: 1.66–2.36; P_adj_ < 0.001) and “other” manifestation at presentation (OR: 2.41, 95% CI: 1.75–3.27; P_adj_ < 0.001). Conversely, infections as the presenting manifestation were less frequent in monogenic CVID cases (OR: 0.67, 95% CI: 0.56–0.81; P_adj_ < 0.001). Neither malignancy nor syndromic features at presentation differed significantly by genetic background (Table 4 C and Fig. 3 B). It should be highlighted that genetic testing was more frequently offered in CVID patients presenting with immune dysregulation (OR: 3.00, 95% CI: 2.66–3.38; P < 0.001), while patients with infectious-predominant presenting phenotypes had less frequently undergone genetic evaluation (OR: 0.72, 95% CI: 0.63–0.82; P < 0.001) (Table S2 C and D).

Current clinical manifestations showed a distinct pattern. In the disease onset–based analysis restricted to patients with recorded follow-up data, pediatric-onset patients were less likely to experience immune dysregulation (OR: 0.58, 95% CI: 0.38–0.87; P_adj_ = 0.019) and malignancies (OR: 0.48, 95% CI: 0.38–0.61; P_adj_ < 0.001) during follow-up, compared with adult-onset patients (Fig. 3 C). Regarding specific immune dysregulation manifestations, pediatric-onset CVID was associated with higher rates of autoimmune endocrinopathies (OR: 8.88, 95% CI: 1.31–380.27) and lower rates of lung involvement including granulomatous–lymphocytic interstitial lung disease (OR: 0.36, 95% CI: 0.20–0.64).

In contrast, current clinical manifestations were not significantly associated with genetic background after multiple-testing correction. Although monogenic CVID demonstrated higher rates of immune dysregulation (OR: 1.60, 95% CI: 0.90–2.87) and lower rates of recurring infection (OR: 0.65, 95% CI: 0.34–1.32) during follow-up, none of these associations reached statistical significance. Regarding specific immune dysregulation manifestations, among 426 patients with available current-manifestation registry data, borderline trends were observed for neurological involvement (OR: 5.56, 95% CI: 1.32–26.89; P_adj_ = 0.055) and enteropathy (OR: 2.50, 95% CI: 1.19–5.03; P_adj_ = 0.055) (Fig. 3 D).

Overall, these analyses indicate that age at disease onset and genetic background capture overlapping but nonidentical dimensions of clinical heterogeneity in CVID, with earlier onset disease linked to extra-immune features and monogenic disease particularly enriched for immune dysregulation phenotypes.

**Figure S1.**
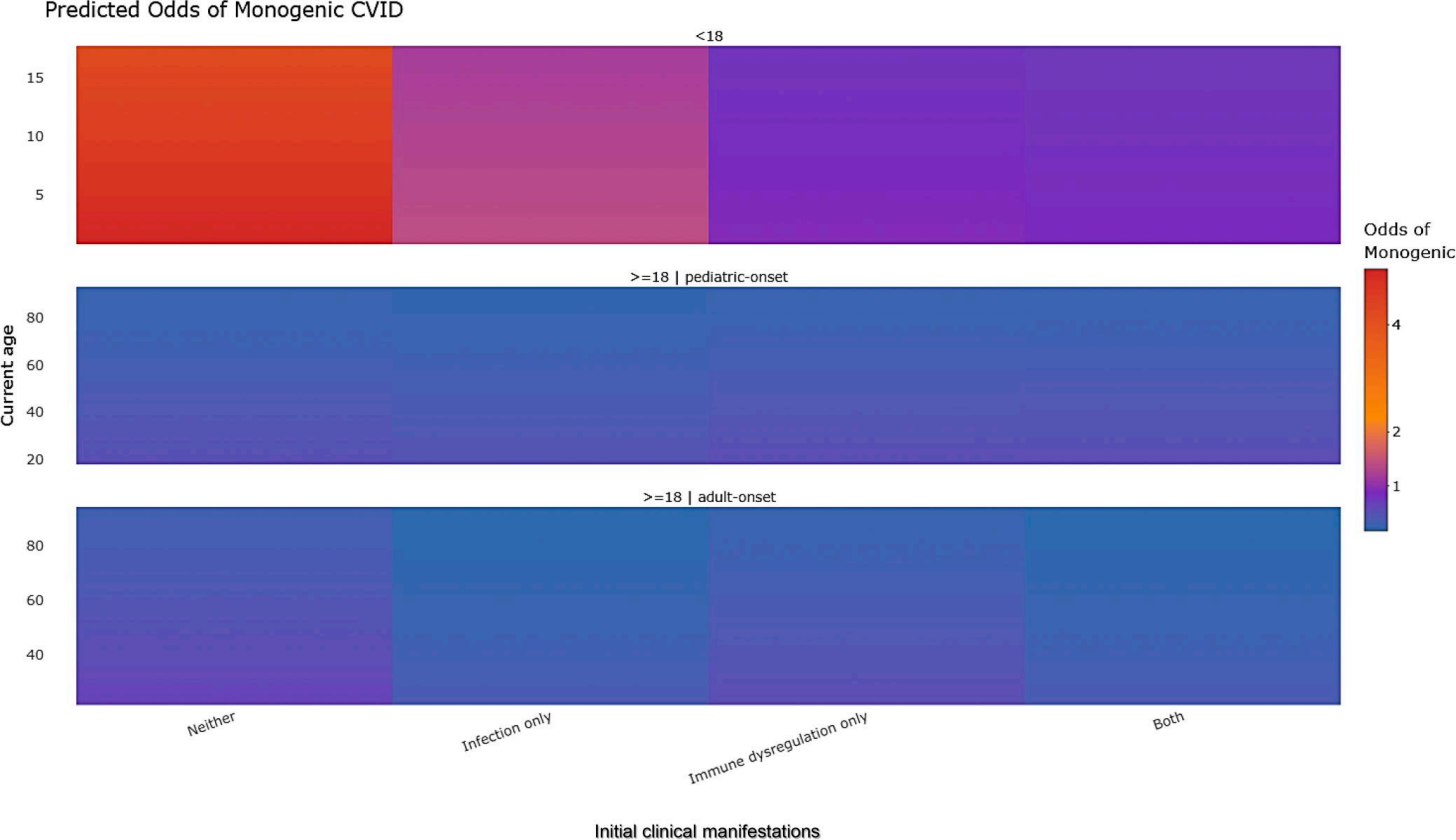
**Heatmap illustrating regression model–derived predicted odds of monogenic CVID across current-age strata and presenting phenotype in ESID-R CVID patients.** Panels distinguish currently pediatric patients, adults with pediatric-onset disease, and adults with adult-onset disease. The highest predicted probabilities were observed among currently pediatric patients with neither infection nor immune dysregulation as the initial manifestation (80.8–83.4%). Interactive HTML version of the heatmap is available at the following link: https://esid.org/html-pages/FigS1_Interactive_HeatMap_CurrentAge_Manifestations.html.

**Figure S2.**
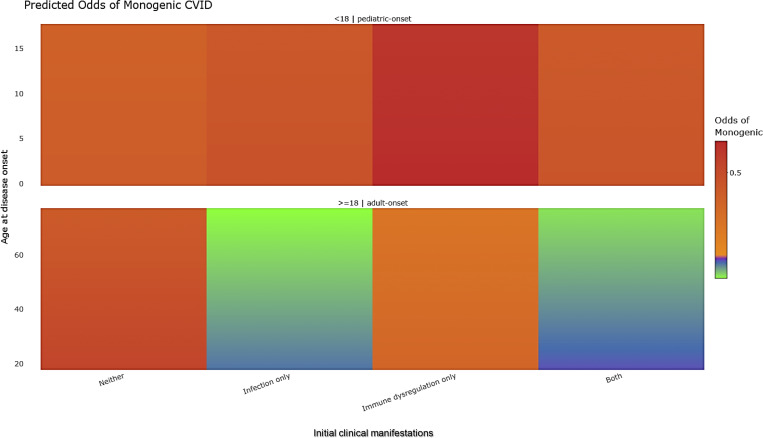
**Heatmap illustrating regression model–derived predicted odds of monogenic CVID across age-at-disease-onset strata and initial manifestation categories in ESID-R CVID patients.** Panels distinguish pediatric-onset and adult-onset disease. Among pediatric-onset patients, predicted probabilities were highest in those presenting with immune dysregulation only (35.6–36.5%). Interactive HTML version of the heatmap is available at the following link: https://esid.org/html-pages/FigS2_Interactive_Interactive_HeatMap_AgeOnset_Manifestations.html.

Regression model–derived predicted probabilities further suggested that monogenic CVID enrichment varied jointly by age and presenting phenotype. In the current-age model, the highest predicted probabilities were observed among currently pediatric patients with neither infection nor immune dysregulation as the initial manifestation (80.8–83.4%), consistent with enrichment of syndromic or other noninfectious presentations in this group (Fig. S1). In the onset-based model, predicted probabilities were highest among pediatric-onset patients presenting with immune dysregulation only (35.6–36.5%), compared with the infection-only (31.7–32.6%), both infection and immune dysregulation (31.4–32.3%), or neither manifestation (30.4–31.2%) (Fig. S2) category.

### Gene enrichment analysis and investigation of broader genetic architecture

Among patients with an identified monogenic defect, the most frequent affected genes were *TNFRSF13B* (*n* = 239), *NFKB1* (*n* = 119), *NFKB2* (*n* = 74), *CTLA4* (*n* = 56), *LRBA* (*n* = 38), *IKZF1* (*n* = 29), *IRF2BP2* (*n* = 26), *ICOS* (*n* = 18), *CD19* (*n* = 11), *PTEN* (*n* = 7), *CR2/CD21* (*n* = 7), *TNFRSF13C* (*n* = 6), *BACH2* (*n* = 5), *ATP6AP1* (*n* = 4), *MS4A1/CD20* (*n* = 3), *TNFSF12* (*n* = 2), *CD81* (*n* = 1), and *TRNT1* (*n* = 1), yielding a total of 646 monogenic CVID cases (Table 1).

**Figure 4.**
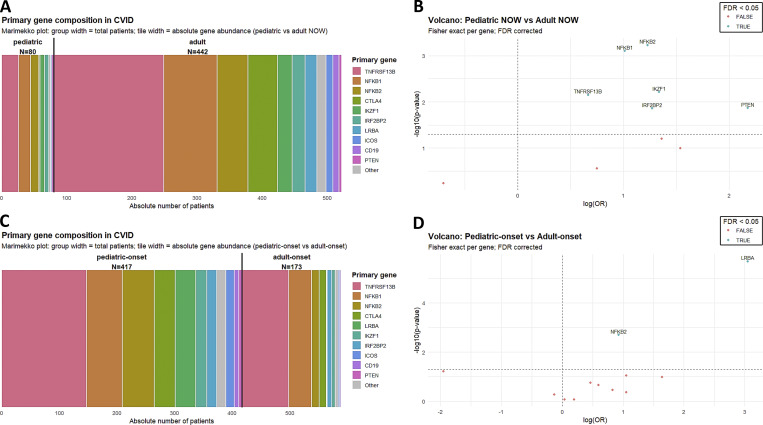
**Visualization of the genetic spectrum of monogenic CVID patients and the results of the gene enrichment analysis. (A and B)** Marimekko plots illustrating the distribution of genetic defects among patients with monogenic CVID between (A) pediatric and adult patients and (B) pediatric-onset and adult-onset cases. **(C and D)** Volcano plots depicting the enrichment of CVID-associated genes in (C) pediatric vs. adult patients and (D) pediatric-onset and adult-onset cases. Regarding Marimekko plots, the width of each colored segment is proportional to the absolute number of patients carrying a defect in the corresponding gene, while the black vertical line markers distinguish between the compared age groups. Genes outside the 10 most frequently affected genes in the overall monogenic CVID cohort were grouped as “Other.” Regarding volcano plots, the x axis represents the log(OR) for enrichment in the pediatric or pediatric-onset group, and the y axis represents -log10(P value). Positive log(OR) values indicate enrichment among pediatric cases. Points are colored according to false discovery rate (FDR) significance. The vertical dashed line indicates log(OR) = 0, corresponding to no enrichment, and the horizontal dashed line indicates the nominal significance threshold. Interactive HTML versions of the Marimekko plots are available at the following links: (A) https://esid.org/html-pages/Fig4A_Interactive_Marimekko_PrimaryGenes_CurrentAge.html; (C) https://esid.org/html-pages/Fig3C_Interactive_Marimekko_PrimaryGenes_AgeOnset.html.

Exploratory gene enrichment analyses refined the age-associated genetic signal within patients with a clinical diagnosis of CVID. Among genetically tested living patients, current pediatric age was associated with enrichment of *TNFRSF13B* (OR: 3.46, 95% CI: 2.15–5.38, P_adj_ < 0.001), *NFKB1* (OR: 4.84, 95% CI: 2.69–8.30, P_adj_ < 0.001), *NFKB2* (OR: 6.01, 95% CI: 2.94–11.49, P_adj_ < 0.001), *IKZF1* (OR: 6.78, 95% CI: 2.42–16.64, P_adj_ = 0.001), *IRF2BP2* (OR: 6.37, 95% CI: 2.07–16.63, P_adj_ = 0.003), and *PTEN* (OR: 15.76, 95% CI: 2.30–93.76, P_adj_ = 0.008). When stratified by age at disease onset, significant enrichment persisted primarily for *NFKB2* (OR: 3.18, 95% CI: 1.71–6.34, P_adj_ < 0.001) and emerged for *LRBA* (OR: 26.52, 95% CI: 4.46–1071.50, P_adj_ < 0.001), suggesting that these genes remained preferentially represented in earlier onset CVID-like disease (Fig. 4).

To investigate broader genetic architecture, an exploratory analysis incorporating variants recorded in the ESID-R “additional genes” field was performed. On this basis, a probable monogenic defect was identified in 838 patients (11.1%) across the cohort, whereas 83 (1.1%) and 35 (0.5%) patients harbored variants in multiple genes suggestive of potential digenic and oligogenic/polygenic architecture, respectively. Exploratory inspection of recurrent multigene combinations showed marked heterogeneity, with nearly all putative digenic/oligogenic combinations observed in only a single patient each. The only recurrent pair was *TNFRSF13B*/*NFKB1*, identified in four patients, whereas the remaining digenic combinations represented isolated pairs and most commonly involved *TNFRSF13B*, *NFKB1*, or *LRBA* paired with another IEI-associated gene (Table S3).

Across the cohort, among patients without an established monogenic diagnosis, the most frequently recorded validated additional genes included *TNFRSF13B* (*n* = 19), *NFKB1* (*n* = 14), *LRBA* (*n* = 12), *CTLA4* (*n* = 8), *NFKB2* (*n* = 8), as well as *TCF3* (*n* = 12), *PIK3CD* (*n* = 10), *NOD2* (*n* = 8), *CR2* (*n* = 7), *VPS13B* (*n* = 7), and *KMT2D* (*n* = 7) (full list in Table S4). The first group reflects variants recorded in genes already known to underlie monogenic CVID-like phenotypes, whereas the latter includes genes more typically linked to other IEI entities. Nevertheless, regarding that these analyses relied on registry-based free-text string annotations rather than systematic variant adjudication, they should be regarded as descriptive and hypothesis-generating rather than equivalent to variant-level genetic classification.

Importantly, inclusion of these additional genes did not generate new robust age-specific enrichment signals, whereas the sparsity of recurrent combinations precluded reproducible phenotype-association analyses. Moreover, age at disease onset was significantly lower in monogenic CVID cases (median 12 years; IQR: 3–24.5; P = 0.003) compared with both nongenetic CVID (median 13 years; IQR: 4–28) and probable oligogenic/polygenic CVID cases (median 13 years, IQR: 5.7–28.4), between which no significant difference was observed. These findings suggest that the major age-dependent signal observed in this cohort is driven primarily by established monogenic disease rather than by a strong and recurrent multigene pattern detectable at the registry level.

## Discussion

This large ESID-R analysis demonstrated a clear age-dependent enrichment of established monogenic diagnoses among patients clinically labeled as CVID, particularly among children and individuals with pediatric disease onset. This pattern was consistent whether patients were stratified by current age or by age at disease onset, and it remained significant after restriction to genetically tested individuals. Importantly, the strongest enrichment of monogenic diagnoses was observed in the earliest onset strata, particularly among patients with disease onset before 4 years of age, supporting the view that early childhood “CVID” frequently represents a genetically defined IEI presenting with a CVID-like phenotype rather than classical, mostly genetically unresolved, CVID (1, 3, 8, 17, 18, 19). Notably, this is concordant with the ESID working definition for clinical diagnosis of CVID (Table S1), which necessitates that the diagnosis is established after the fourth year of life, thereby formally acknowledging the diagnostic instability of CVID in early childhood (4).

Accordingly, the youngest age strata require cautious interpretation. In keeping with ESID and other diagnostic criteria, CVID is generally not considered a stable diagnosis before 4 years of age, and very young children with severe or immune dysregulation phenotypes may be preferentially reclassified into other IEI categories after genetic testing (4). Therefore, the <4-year current-age subgroup should be interpreted descriptively only. Importantly, disease onset before 4 years of age should not be equated with CVID diagnosis before 4 years of age, but indicates that symptoms later contributing to a CVID or CVID-like registry diagnosis began in early childhood. Consistent with this interpretation, a sensitivity analysis excluding patients with clinical CVID diagnosis before 4 years showed that pediatric-onset disease remained significantly associated with monogenic CVID in both the whole-cohort and the genetically tested subgroup.

This distinction matters conceptually, as CVID has long functioned as an umbrella or provisional clinical diagnosis that is frequently revised once a specific genetic etiology is identified (4, 5, 20). Furthermore, in adults, CVID serves as a residual category that remains genetically unresolved, with probable unexplained polygenic or multifactorial disease, despite increasing molecular insight, whereas in children, the CVID label appears substantially less coherent (2, 5, 20, 21). The younger the patient—and especially the earlier the onset—the greater the prior probability that a CVID label is capturing the early presentation of a genetically defined IEI, especially if it presents with immune dysregulation features, as supported by our analyses (8, 22, 23, 24, 25). This is clinically relevant because the differential diagnosis of pediatric CVID-like disease is broad, including evolving monogenic IEI, such as combined immunodeficiencies, primary immune regulatory disorders or syndromic IEI, and transient hypogammaglobulinemia of infancy and secondary causes of hypogammaglobulinemia, particularly those related to B cell–depleting medications (1). Our findings support viewing “pediatric CVID” less as a clearly delimited disease entity and more as a provisional phenotype-based label that warrants ongoing genetic evaluation and longitudinal reassessment. This view aligns with current literature highlighting the diagnosis of CVID in childhood is inherently challenging due to developmental immunological immaturity, overlap with other IEIs, and the increasing recognition of monogenic immunological defects (1, 12, 26).

The clinical phenotype data add nuance to this interpretation. In our cohort, monogenic CVID was significantly associated with immune dysregulation at presentation, whereas the genetically unresolved group more often retained the classic infection-predominant profile. These findings are consistent with previous studies demonstrating that immune dysregulation—including autoimmunity and lymphoproliferation—is a hallmark feature of many genetically defined IEIs (22, 27, 28, 29). However, immune dysregulation itself was not enriched among pediatric patients in our age-stratified analyses, being more frequent in adult-onset cases. This inconsistency could be attributed to ascertainment and classification bias: children presenting with early-onset immune dysregulation may be more likely to undergo genetic testing and, once a causal defect is identified, to be reclassified into another IEI category rather than to remain captured within the CVID dataset (25, 30, 31). This may explain the reduced representation of immune dysregulation phenotypes within the pediatric CVID subgroup that remained available for analysis. Moreover, the lack of association between monogenic CVID and immune dysregulation features during follow-up may be attributed to limited registry completeness of the “current manifestations” fields that were introduced only in December 2024.

Gene enrichment analyses further refined the age-dependent genetic architecture of CVID, demonstrating that the pediatric signal was mainly driven by monogenic defects, rather than by an emerging multigenic signature. Several genes, including *TNFRSF13B*, *NFKB1*, *NFKB2*, *IKZF1*, *IRF2BP2*, and *PTEN*, were enriched in the pediatric CVID subgroup, whereas in the onset-based analysis, the most consistent associations with pediatric-onset emerged primarily for *NFKB2* and *LRBA*, both of which are strongly linked in the literature to early-onset disease presentations (32, 33). Many of these genes are involved in immune regulatory pathways and lymphocyte signaling, consistent with the immune dysregulation phenotypes observed in monogenic CVID-like disease (3, 5). Furthermore, exploratory analyses among CVID patients without an established monogenic diagnosis identified several recurrent candidate genes, including *TCF3*, *PIK3CD*, and *KMT2D*, which have been associated with immune dysregulation features in literature (34, 35, 36). However, these recurrent genes are best interpreted as biologically plausible and need variant-level functional confirmation.

Beyond the enrichment of established monogenic diagnoses in CVID-like patients, our exploratory analyses suggested that a small subset of patients may not conform to a strictly monogenic model. Potential digenic or oligogenic/polygenic constellations were identified in ∼1.6% of the cohort based on registry-recorded additional-gene annotations. This figure should be interpreted as the frequency of such annotations in the ESID-R dataset, rather than as the true prevalence of multiple rare variants detected by genetic testing in CVID. These observations support the concept that some patients labeled as CVID may lie along a continuum between clearly defined monogenic IEI and more complex multigenic immune dysregulation (1, 3, 9, 37). However, almost all putative digenic pairs were observed only once across the cohort, arguing against a strong and shared multigenic architecture. Instead, these data indicate that broader genetic contributions, where present, are likely to be individually variable and with inconsistent genotype–phenotype correlation. Notably, *TNFRSF13B* emerged as the most frequent recurring primary gene within putative digenic pairs, being consistent with its reported involvement in epistatic interactions (38, 39). Overall, these findings are more compatible with a model of occasional modifier or epistatic effects superimposed on a heterogeneous CVID genetic background than with a reproducible multigenic architecture that explains the age-dependent signal observed in our cohort. In this context, multigenic inheritance may become relatively more relevant in late-onset or adult CVID, where the burden of clearly established monogenic disease is lower, although this remains an inference rather than a demonstrated age-specific pattern in our data. Accordingly, these hypothesized multigenic constellations should be interpreted cautiously and as hypothesis-generating, since they were derived from registry annotations rather than systematic variant adjudication and functional confirmation.

Taken together, these findings raise an important conceptual question: does CVID exist in children? Our data do not justify abolishing the term outright, as not all pediatric cases with CVID-like phenotype were attributed to an identifiable monogenic defect. However, they do indicate that the CVID diagnosis should be applied with substantially greater caution in early-onset disease, particularly in children presenting before 4 years of age or with features of immune dysregulation, including autoimmune cytopenias or other organ-specific autoimmunity, lymphoproliferation, enteropathy, granulomatous disease, and rheumatological and/or autoinflammatory manifestations. In such settings, CVID may be better regarded as a provisional clinical designation, pending iterative genetic investigation, and repeated clinical and immunological reevaluation, rather than as a definitive endpoint diagnosis. Given that a substantial proportion of pediatric cases represent genetically defined IEI, the CVID label may obscure the underlying disorder and delay appropriate genetic evaluation. Our findings therefore support systematic genetic testing in early-onset antibody deficiency and/or immune dysregulation. Although current ESID diagnostic criteria allow the diagnosis of CVID in children, they also emphasize exclusion of defined IEI (4). In this context, our results suggest that age should be considered more explicitly in future diagnostic frameworks, as pediatric cases show a markedly higher likelihood of underlying monogenic disease, including many disorders or manifestations that are amenable to targeted immune-modifying drugs (28).

This study has several limitations inherent to registry-based analysis. Firstly, it has to be acknowledged that the ESID-R preserved physician-entered clinical diagnoses and does not overrule them after molecular results become available, unless the treating physician decides to reclassify the patient (13). Consequently, a proportion of patients harboring monogenic defects may have remained classified as CVID, which explains both the enrichment of *LRBA* mutations in pediatric-onset disease and the presence of variants in genes such as *TCF3*, *PIK3CD*, and *KMT2D*, associated with other well-defined IEI. Conversely, some of the clearest monogenic pediatric cases may already have been reclassified out of the CVID cohort before this analysis, implying that the present cohort reflects a population already shaped by partial genotype-driven diagnostic sorting (4). Secondly, testing and ascertainment bias must be considered, as children with early-onset, severe, syndromal, familial, or immune dysregulation phenotypes are more likely to undergo genetic testing than adults with milder infection-predominant disease, which is reflected also by our results (25). Accordingly, whole-cohort estimates should not be interpreted as genetic diagnostic yield, but rather as registry-level indicators of the burden of genetically defined IEI among patients still recorded under a clinical diagnosis of CVID. However, the persistence of the age-dependent enrichment of monogenic disease in the genetically tested subset suggests that differential testing alone does not account for the findings. Finally, exploratory analyses of digenic or oligogenic architecture were based on physician-entered free-text registry annotations of gene names only rather than systematic variant-level adjudication, CADD-score filtering, raw sequencing reanalysis, or functional validation and should therefore be interpreted as hypothesis-generating rather than as evidence for confirmed multigenic inheritance. Variant-level information is not systematically captured in the ESID-R, in part due to data-protection considerations under General Data Protection Regulation (GDPR). Therefore, additional-gene entries may include variants of unknown significance, candidate variants, potential modifier variants, or other physician-entered genetic annotations, and should be interpreted as exploratory registry annotations rather than evidence for established digenic or oligogenic inheritance. Nevertheless, the large sample size and the multicenter nature of the ESID-R provide robust real-world evidence regarding the genetic architecture of CVID.

These limitations also point toward constructive next steps. A structured ESID-R re-adjudication study of genotype-positive patients still labeled clinically as CVID could help clarify whether such cases should remain under a CVID-like designation or be reassigned to a more specific IEI category, while preserving transparency and the clinical judgment of the treating physician. More broadly, future ESID-R and classification frameworks may benefit from a dual-axis diagnostic classification model, recording both a clinical and a molecular diagnosis in parallel, even when they are not fully aligned. This strategy may better capture the phenotypic overlap and the diagnostic evolution that characterize IEI, particularly in childhood, where a CVID-like phenotype may precede the final molecular diagnosis.

In conclusion, this ESID-R analysis delineates an age-dependent map of genetic architecture and clinical manifestations in CVID, demonstrating that a substantial proportion of pediatric cases labeled as CVID likely represent genetically defined IEI, especially those with the earliest onset. These findings challenge the validity of CVID as a stable diagnostic entity in childhood and highlight the need for systematic genetic evaluation in early-onset CVID-like disease and/or immune dysregulation at presentation, while recognizing that genetic testing remains clinically relevant in adult CVID for diagnosis, prognosis, family counseling, and targeted management. Future diagnostic or classification frameworks should incorporate age more explicitly to better distinguish genetically defined IEI from genetically unresolved CVID across the full age spectrum.

## Materials and methods

In this retrospective study, we analyzed patients recorded in the ESID-R under the current diagnosis of CVID as entered by the treating physician, requiring the fulfillment of the ESID clinical diagnostic criteria for CVID (Table S4) (4), regardless of the presence of an identified genetic defect. Registered patients or their parents/legal representatives provided written informed consent. The study was conducted with approval from the ESID-R Steering Committee and local ethics committees (on the basis of the latest amendment from Institutional Review Board 00002556/24-334ex11/12).

Data extraction included demographic variables, current age, age at disease onset, genetic status, and initial and current clinical manifestations. Current-age categories were defined using January 17, 2026, as the reference date. Genetic status included information recorded in the ESID-R “current IEI diagnosis” field, as well as the additional genes section.

Monogenic CVID was defined as a current IEI diagnosis with an underlying genetic defect associated with a CVID-like phenotype (*ATP6AP1*, *BACH2*, *TNFRSF13C/BAFFR*, *CD19*, *MS4A1/CD20*, *CR2/CD21*, *CD81/TAPA1*, *CTLA4*, *ICOS*, *IKZF1*, *IRF2BP2*, *LRBA*, *MOGS*, *NFKB1*, *NFKB2*, *PTEN*, *TNFRSF13B/TACI*, *TRNT1*, *TTC37/SKIC3*, *TNFSF12/TWEAK*). It should be highlighted that some of the aforementioned genes (*CTLA4*, *LRBA*) are currently classified in different IUIS categories, namely, among diseases of immune dysregulation or primary immune regulatory disorders (12). This discrepancy is attributed to the fact that, historically, the ESID-R allowed physicians to enter a clinical diagnosis of CVID based on clinical criteria and, subsequently, record a genetic defect, even if the gene had been or was later assigned to a distinct IEI entity. Consequently, certain genetically defined IEIs remain recorded under CVID in the ESID-R dataset. To preserve the original physician-reported diagnosis and avoid retrospective system-caused reclassification, these cases were retained in the analysis. Since the latest iteration of the ESID-R platform, however, genetic diagnoses are prioritized and automatically determine the diagnosis classification, but physicians may still assign different clinical diagnoses depending on the presenting phenotype.

Age was analyzed using two complementary approaches: (1) current-age category (pediatric [<18 years] vs. adult [≥18 years]); and (2) age at disease onset (pediatric-onset [<18 years] vs. adult-onset [≥18 years]). Additional stratified analyses were performed across predefined age subgroups (<4, 4–7, 8–11, 12–17, 18–25, and >25 years of age).

Associations between age categories, genetic status, and presenting clinical manifestations were evaluated using contingency analyses, ORs, and Pearson’s x^2^ or Fisher’s exact test, as appropriate. Mosaic and Marimekko plots were used to visualize associations between age categories and monogenic CVID status, whereas volcano plots were used to illustrate enrichment of CVID-associated genes in pediatric versus adult cases.

To explore the potential contribution of secondary variants and possible polygenic inheritance, a string-mining approach was applied to the additional genes free-text field in the ESID-R dataset. Gene names reported in these entries were extracted, standardized, and analyzed to identify recurrent candidate variants among patients without an established monogenic diagnosis. Additional genes refer to further physician-entered genetic annotations recorded in the ESID-R, and may therefore include variants of uncertain significance, candidate variants, potential modifier variants, or other physician-entered genetic annotations, as variant-level information is not systematically captured in the ESID-R due to data-protection considerations under GDPR.

Data cleaning and all statistical analyses were performed using R programming language version 4.1.3 (40).

### Declaration of generative AI

The authors used ChatGPT, GPT-5.5 Thinking model, for partial text editing to improve readability; they then reviewed and edited the text and took full responsibility for the content.

### Online supplemental material

In this manuscript, the additional material provides additional context for the definitions, testing patterns, and exploratory genetic architecture of the ESID-R CVID cohort. Figs. S1 and S2 present regression-derived heatmaps of predicted monogenic CVID probability across age strata (Fig. S1: current age; Fig. S2: age at disease onset) and initial manifestations, with interactive HTML versions available through the ESID website. Table S1 summarizes the ESID-R working definition for a clinical diagnosis of CVID. Table S2 reports associations between genetic testing and age or presenting phenotype, highlighting higher testing rates in pediatric patients, pediatric-onset disease, and immune dysregulation. Tables S3 and S4 describe exploratory additional-gene annotations, including digenic/oligogenic entries and candidate genes recorded in patients without an established monogenic diagnosis, respectively.

## Supplementary Material

Table S1 shows ESID-R working definition for a clinical diagnosis of CVID (modified from [4]).

Table S2 shows associations between genetic testing status and (A) current-age category; (B) age-at-disease-onset category; (C) presence of infections as initial manifestation; and (D) presence of immune dysregulation as initial manifestation.

Table S3 shows most common digenic/oligogenic pairs by primary gene in our CVID cohort.

Table S4 shows additional genes identified in patients without an established monogenic diagnosis.

Table S5 lists ESID Registry Working Party members.

## Data Availability

Patient-level data are not publicly available due to privacy rights. Data underlying the figures in this manuscript may be available upon reasonable request from the corresponding author. Detailed interactive versions of selected figures, including all panels of Fig. 2 and panels A and C of Fig. 4, are available on the ESID website, with links provided in the corresponding figure legends.
