# Supplementary material for: Does CVID exist in children? A genetic architecture and manifestation map derived from 7,525 patients
Source: J Hum Immun. 2026 Jul 23;2(5):e20260091. doi: 10.70962/jhi.20260091 (PMC13394009; doi:10.70962/jhi.20260091)
Supplement: Table S1 — shows ESID-R working definition for a clinical diagnosis of CVID (modified from [4]). [file jhi_20260091_tables1.docx]

**Supplementary Table S1. ESID-R working definition for a clinical diagnosis of CVID** (modified from Seidel et al.,*J Allergy Clin Immunol Pract*. 2019)

| **Clinical Criteria for a probable diagnosis of CVID *(ALL criteria should be fulfilled)*** | |
| --- | --- |
| **Criteria** | **Definition** |
| **CVID-like Clinical Features or  Positive Family History**  **AND** | At least one of the following must be present:   - Increased susceptibility to infection - Autoimmune manifestations - Granulomatous disease - Unexplained polyclonal lymphoproliferation - Affected family member with antibody deficiency |
| **Hypogammaglobulinemia**  **AND** | Marked decrease of IgG and IgA, with or without low IgM levels  *(defined as <2 SD below age-adjusted normal levels; measured at least twice)* |
| **Functional B-cell Deficit**  **AND** | At least one of the following:   - Poor antibody response to vaccines and/or absent isohemagglutinins - Low switched memory B cells *(defined as <70% of age-related normal value)* |
| **Exclusion of  secondary causes**  **AND** | Secondary causes of hypogammaglobulinemia must be excluded  *(e.g., infection, protein loss, medication, or malignancy)*, as well as other well defined monogenic IEI |
| **Minimal Age Requirement**  **AND** | Diagnosis should be established after the fourth year of life  *(although symptoms may be present earlier)* |
| **Exclusion of profound  T-cell deficiency** | No evidence of profound T-cell deficiency, defined as 2 of the following 3 criteria:   - CD4+T-cells *(cells/µL)*: 2–6 years <300; 6–12 years <250; >12 years <200 - Naive CD4+T-cells *(%)*: 2–6 years <25%; 6–16 years <20%; >16 years <10% - Absent T-cell proliferation |
