# Supplementary material for: Does CVID exist in children? A genetic architecture and manifestation map derived from 7,525 patients
Source: J Hum Immun. 2026 Jul 23;2(5):e20260091. doi: 10.70962/jhi.20260091 (PMC13394009; doi:10.70962/jhi.20260091)
Supplement: Table S2 — shows associations between genetic testing status and (A) current-age category; (B) age-at-disease-onset category; (C) presence of infections as initial manifestation; and (D) presence of immune dysregulation as initial manifestation. [file jhi_20260091_tables2.docx]

**Supplementary Table S2. Associations between genetic testing status and (A) current age category; (B) age at disease onset category; (C) presence of infections as initial manifestation; and (D) presence of immune dysregulation as initial manifestation.**

| A) Association between current age category and genetic testing status | | | |
| --- | --- | --- | --- |
| Current age | Genetically tested | Not genetically tested | Total |
| Pediatric | 142 (56.6%) | 109 (43.4%) | 251 |
| Adult | 1642 (31.4%) | 3583 (68.6%) | 5225 |
| Total | 1784 (32.6%) | 3692 (67.4%) | 5476 |
| OR (pediatric *vs* adult): 2.84 (95%CI: 2.20–3.67), *p*<0.001, Cramer’s V=0.112 | | | |
|  | | | |
| B) Association between age at disease onset and genetic testing status | | | |
| Age at onset | Genetically tested | Not genetically tested | Total |
| Pediatric-onset | 1303 (31.2%) | 2877 (68.8%) | 4180 |
| Adult-onset | 743 (24.3%) | 2311 (75.7%) | 3054 |
| Total | 2046 (28.3%) | 5188 (71.7%) | 7234 |
| OR (pediatric-onset *vs* adult-onset): 1.41 (95%CI: 1.27–1.57), *p*<0.001, Cramer’s V=0.075 | | | |
|  | | | |
| C) Association between infections at presentation and genetic testing status | | | |
| Initial infectious manifestation | Genetically tested | Not genetically tested | Total |
| Yes | 1687 (31.1%) | 3737 (68.9%) | 5424 |
| No | 458 (38.5%) | 732 (61.5%) | 1190 |
| Total | 2145 (32.4%) | 4469 (67.6%) | 6614 |
| OR (infections: yes *vs* no): 0.72 (95%CI: 0.63–0.82), *p*<0.001, Cramer’s V=0.061 | | | |
|  | | | |
| D) Association between immune dysregulation at presentation and genetic testing status | | | |
| Initial immune dysregulation | Genetically tested | Not genetically tested | Total |
| Yes | 766 (52.3%) | 698 (47.7%) | 1464 |
| No | 1379 (26.8%) | 3771 (61.5%) | 5150 |
| Total | 2145 (32.4%) | 4469 (67.6%) | 6614 |
| OR (immune dysregulation: yes *vs* no): 3.00 (95%CI: 2.66–3.38), *p*<0.001, Cramer’s V=0.227 | | | |
|  | | | |
