# Supplementary material for: Does CVID exist in children? A genetic architecture and manifestation map derived from 7,525 patients
Source: J Hum Immun. 2026 Jul 23;2(5):e20260091. doi: 10.70962/jhi.20260091 (PMC13394009; doi:10.70962/jhi.20260091)
Supplement: Table S3 — shows most common digenic/oligogenic pairs by primary gene in our CVID cohort. [file jhi_20260091_tables3.docx]

**Supplementary Table S3. Most common digenic/oligogenic pairs by primary gene in our CVID cohort.**

| **Primary gene** | **Secondary gene(s)** | **Patients (n)** |
| --- | --- | --- |
| ***CD19*** | *LAT* | 1 |
|  | ***NFKB1*** | 1 |
| ***IKZF1*** | ***LRBA*** | 1 |
| ***IRF2BP2*** | *ANKRD11* | 1 |
|  | *KMT2D* | 1 |
| ***LRBA*** | *CTLA4* | 1 |
|  | *IL4I1* | 1 |
|  | *PIK3CD* | 1 |
|  | *PLCG2* | 1 |
| ***NFKB1*** | *FASLG* | 1 |
|  | *NOD2* | 1 |
|  | *PRDM1,* ***TNFRSF13B*** | 1 |
|  | *RAG2, TNFSF10* | 1 |
|  | *TCF3* | 1 |
|  | ***TNFRSF13B*** | 1 |
|  | *TNFRSF13C* | 1 |
| ***NFKB2*** | *SH3BP2* | 1 |
| ***TNFRSF13B*** | ***NFKB1*** | 2 |
|  | *CASP8* | 1 |
|  | *CD19* | 1 |
|  | *COL14A1* | 1 |
|  | *COL5A2* | 1 |
|  | *CTC1* | 1 |
|  | *CTSC* | 1 |
|  | *CXCR4* | 1 |
|  | *DES* | 1 |
|  | *MBL2* | 1 |
|  | *MSH6* | 1 |
|  | *NFKB2* | 1 |
|  | *NOTCH2* | 1 |
|  | *PRKCD* | 1 |
|  | *RELA* | 1 |
|  | *STAT1* | 1 |
|  | *STX11* | 1 |
|  | *FLG, NLRP3* | 1 |
|  | *NOD2, STXBP2* | 1 |
|  | *DNAH11, LYST, SLX4, STIM1, TNFAIP3* | 1 |
| Primary genes refer to genes considered by the reporting center to explain the patient’s genetically defined IEI diagnosis and were used in this analysis to classify patients as having monogenic CVID. Additional genes refer to further physician-entered genetic annotations recorded in the ESID-R. These additional-gene entries were not uniformly adjudicated across centers as pathogenic, likely pathogenic, or variants of uncertain significance according to ACMG/AMP criteria, and variant-level information was not systematically available in the ESID-R due to data-protection considerations under GDPR. Additional genes may therefore include variants of uncertain significance, candidate variants, potential modifier variants, or other physician-entered genetic annotations, and should be interpreted as exploratory registry annotations rather than confirmed digenic or oligogenic disease mechanisms. | | |
