# Supplementary material for: Does CVID exist in children? A genetic architecture and manifestation map derived from 7,525 patients
Source: J Hum Immun. 2026 Jul 23;2(5):e20260091. doi: 10.70962/jhi.20260091 (PMC13394009; doi:10.70962/jhi.20260091)
Supplement: Table S4 — shows additional genes identified in patients without an established monogenic diagnosis. [file jhi_20260091_tables4.docx]

**Supplementary Table S4. Additional genes identified in patients without an established monogenic diagnosis. (A) Secondary genes corresponding to genes already included in the primary monogenic CVID gene list. (B) Secondary genes not included in the primary gene list. Counts (n) are unique patients. Denominator for percentages = all patients with no known genetic defect (NKGD; n = 6,879).**

| **A. Genes of the primary list** | **n** | **% of NKGD** | **B. Genes associated with other IEI** | **n** | **% of NKGD** |
| --- | --- | --- | --- | --- | --- |
| *TNFRSF13B* | 19 | 0.3 | *TCF3* | 12 | 0.2 |
| *NFKB1* | 14 | 0.2 | *PIK3CD* | 10 | 0.1 |
| *LRBA* | 12 | 0.2 | *NOD2* | 8 | 0.1 |
| *CTLA4* | 8 | 0.1 | *CR2* | 7 | 0.1 |
| *NFKB2* | 8 | 0.1 | *KMT2D* | 7 | 0.1 |
| *BACH2* | 4 | 0.1 | *VPS13B* | 7 | 0.1 |
| *IRF2BP2* | 4 | 0.1 | *CARD11* | 6 | 0.1 |
| *IKZF1* | 3 | 0.0 | *PIK3R1* | 6 | 0.1 |
| *TNFRSF13C* | 2 | 0.0 | *KMT2A* | 5 | 0.1 |
| *CD19* | 1 | 0.0 | *RTEL1* | 5 | 0.1 |
| *CD81* | 1 | 0.0 | *TBX1* | 5 | 0.1 |
| *ICOS* | 1 | 0.0 | *NLRP1* | 4 | 0.1 |
| *MS4A1* | 1 | 0.0 | *TRAF3* | 4 | 0.1 |
| *TNFSF12* | 1 | 0.0 | *UNC13D* | 4 | 0.1 |
| *TRNT1* | 1 | 0.0 | *ATM* | 3 | 0.0 |
