# Supplementary material for: Does CVID exist in children? A genetic architecture and manifestation map derived from 7,525 patients
Source: J Hum Immun. 2026 Jul 23;2(5):e20260091. doi: 10.70962/jhi.20260091 (PMC13394009; doi:10.70962/jhi.20260091)
Supplement: Table S5 — lists ESID Registry Working Party members. [file jhi_20260091_tables5.docx]

**ESID registry working party / collaborators list (last name, first name):** Abd Elaziz, Dalia; Abdelkader, Sohilla Lofty M.; Abitbol, Avigaelle; Abolhassani, Hassan; Abraham, Rinchu; Abrahim, Lalash; Abuzakouk, Mohamed; Accardo, Pietro Andrea; Achir Moussouni, Nabila; Afonso, Veronica; Agyeman, Philipp; Ahlmann, Martina; Aiuti, Alessandro; Akl, Abla; Aksu, Güzide; Al-Dehni, Fadi; Albers, Kim; Albert, Michael H.; Alecsandru, Meda Diana; Aleinikova, Olga; Aleshkevich, Svetlana; Alkady, Radwa Salah Eldeen Youssif; Allende, Luis; Alligon, Mickaël; Allwood, Zoe; Alsina, Manrique de Lara Laia; Amadi, Chibuonu Judith; Ambrosch-Barsoumian, Daniela; Ameshofer, Lisa; Amour, Kenza; Anadol, Evrim; Ananthachagaran, Ariharan; Andrejevic, Sladjana; Andriamanga, Chantal; Andris, Julia; Andritschke, Karin; Angelini, Federica; Ankermann, Tobias; Anyango, Yvonne; Apel, Katrin; Arami, Siamak; Ardeniz, Ömür; Arkwright, Peter; Arlabosse, Tiphaine; Arnold, Karina; Ascherl, Rudolf; Assam, Najla; Assia-Batzir, Nurit; Atschekzei, Faranaz; Aumann, Sybille; Aumann, Volker; Aurivillius, Magnus; Ausserer, Bernd; Avcin, Tadej; Aydemir, Sezin; Aygören-Pürsün, Emel; Ayvaz, Deniz N. Cagdas; Azhar, Alisa; Azzari, Chiara; Bach, Perrine; Bachmann, Sophie; Bader, Peter; Badolato, Raffaele; Bahal, Sameer; Bakhtiar, Shahrzad; Bancé, Renate; Bangeas, Athanasios; Bangs, Catherine; Barfusz, Katerina; Baris, Safa; BarlanIsil, B; Bartsch, Michaela; Baselli, Lucia Augusta; Batlle-Maso, Laura; Baumann, Helge; Baumann, Ulrich; Baumeister, Veronika; Baxendale, Helen; Bazen, Suzanne; Beaurain, Beatrice; Beauté, Julien; Bechar-Makhloufi, Mounia; Beck, Norbert; Becker, Brigitta; Becker, Christian; Behrends, Uta; Beider, Renata; Beier, Rita; Belkacem, Amel; Belke, Luisa; Bellert, Sven; Belohradsky, Bernd H.; Ben-Bouzid, Aouatef; Benjamin, Kimberley; Benoît, Vincent; BenSlama, Lilia; Berdous-Sahed, Thamila; Bergils, Jan; Berglöf, Anna; Bergman, Peter; Bernat-Sitarz, Katarzyna; Bernatoniene, Jolanta; Bernatowska, Ewa; Bernbeck, Benedikt; Bertolini, Elena; Bethune, Claire; Beuckmann, Kai; Bhole, Malini; Biegner, Anika-Kerstin; Bielack, Stefan; Bienemann, Kirsten; Bigl, Arndt; Bigorgne, Amélie; Bijl, Marc; Binder, Nadine; Bitzenhofer-Grüber, Michaela; Blanchard Rohner, Geraldine; Blank, Dagmar; Blattmann, Claudia; Blau, Julia; Blaziene, Audra; Blazina, Stefan; Bloomfield, Markéta; Blume, Roswitha; Boardman, Barbara; Bode, Sebastian; Boelens, Jaap-Jan; Boesecke, Christoph; Bogaert, Delfien; Bogner, Johannes; Bohynikova, Nadezda; Booth, Claire; Bordon, Victoria; Borkhardt, Arndt; Borte, Michael; Borte, Stephan; Bossaller, Lukas; Bossard, Madeleine; Boucherit, Soraya; Boutros, Jeannette; Bouzoumita, Amira; Bower, Clare; Boyarchuk, Oksana; Boyman, Onur; Boztug, Kaan; Braschler, Thomas; Bravo, Sophie; Bredius, Robbert; Bright, Philip; Brito de Azevedo Amaral, Carolina; Brodszki, Nicholas; Brodt, Grit; Brolund, Allan; Brosselin, Pauline; Brummel, Bastian; Brun-Schmid, Sonja; Brunner, Jürgen; Bruns, Roswitha; Buchta, Christina; Buck, Dietke; Buckland, Matthew; Burdach, Stefan; Burns, Siobhan; Burton, Janet; Byrne, Derval; Börries, Melanie; Bücker, Aileen; Bührlen, Martina; Caminal, Luis; Cancrini, Caterina; Candotti, Fabio; Canessa, Clementina; Cannon, Jessica; Cant, Andrew J; Cantoni, Nathan; Capilna, Brindusa; Caracseghi, Fabiola; Caragol, Isabel; Carbone, Javier; Carrabba, Maria; Casanova, Jean-Laurent; Chamberlain, Latanya; Chandra, Anita; Chantrain, Christophe; Chapel, Helen; Charisi, Konstantina; Chassot, Julie; Chee, Ronnie; Cherian, Deepti; Chinello, Matteo; Chopra, Charu; Chovancova, Zita; Christmann, Martin; Chrzanowska, Krystyna; Ciznar, Peter; Claes, Karlien; Classen, Carl Friedrich; Classen, Martin; Cochino, Alexis-Virgil; Collins, Lee; Condliffe, Alison M.; Condy, Damaris; Corbacioglu, Selim; Cordeiro, Ana Isabel; Cordier Wynar, Donatienne; Core, Claire; Costes, Laurence; Coulter, Tanya; Courteille, Virginie; Cristina, Maria; Cucuruz, Maria; Dabrowska-Leonik, Nel; Daly, Mary Louise; Daniel, Claudia-Sabrina; Danieli, Maria Giovanna; Darroch, James; Davies, Graham; De Gracia Roldan, Javier; de Nadai, Narimene; de Schutter, Iris; De Vergnes, Nathalie; de Vries, Esther; de Witte, Josine; deBaets, Frans; Debert, Theo; DeBoeck, Christiane; Defila, Corina; Deimel, Judith; Delaplace, Diane; Dellepiane, Rosa Maria; Dellert, Nelli; Delliera, Laura; Delor, Anita; Demel, Ulrike; Dempster, John; den Os, M.M.; Dengg, Rosmarie; Desisa, Sora Asfaw; Desta, Alexandra; Detkova, Drahomira; Dewerchin, Maite; Dieli Crimi, Romina; Dilloo, Dagmar; Dimitriou, Florentia; Dinges, Sarah Svenja; Dinser, Jasmin; Dipani, Nabila; Dirks, Johannes; Dittrich, Anna-Maria; Djermane, Lylia; Dogru, Yagmur; Dogu Esin, Figen; Dombrowski, Angelika; Dominguez Escobar, Julia; Drabe, Camilla Heldbjerg; Drerup, Susann; Drexel, Barbara; Driessen, G.J.A.; Dudoit, Yasmine; Duppenthaler, Andrea; Dähling, Mandy; Döring, Michaela; Dückers, Gregor; Ebetsberger-Dachs, Georg; Ecser, Mate Barnabas; Edgar, J. David; Eekman, Maartje; Ehl, Stephan; Ehrat, Rosanna; Eisl, Eva; Ekwall, Olov; El Hawary, Rabab; El-Helou, Sabine M.; El-Marsafy, Aisha; Elbe, Sarina; Elcombe, Suzanne; Eldash, Alia; Ellerbroek, P.M.; Elling, Roland; Elliott, Jane; Emilie Nielsen, Nynne; Engelhardt, Angelika; Ernst, Diana; Ersoy, Fügen; Esper, Stefanie; Esteves, Isabel; Etzioni, Amos; Exley, Andrew; Faber, Martin; Fabio, Giovanna; Fahrni, Gaby; Faletti, Laura Eva; Farber, Claire-Michele; Farela Neves, João; Faria, Emilia; Farkas, Henriette; Farmaki, Evangelia; Faßhauer, Maria; Fasth, Anders; Faustmann, Stefanie; Fecker, Gisela; Feighery, Conleth; Feiterna-Sperling, Cornelia; Fernandez-Cruz, Perez Eduardo; Ferreira Concalo, Cordeiro; Ferster, Alice; Feuchtinger, Tobias; Feyen, Oliver; Finke, Daniela; Fischer, Alain; Fisher, Bethany; Fitter, Sigrid; Flaschberger, Stefan; Fleckenstein, Lucia; Fontana, Adriano; Forino, Concetta; Franchet, Nora; Freitag, Dagmar; Frey, Urs P.; Frick, Hannah Margarete; Friedel, Elisabeth; Friedrich, Wilhelm; Frisch, Barbara; Frischknecht, Lukas; Fritsch, Sissi; Fritzemeyer, Stephanie; Fätkenheuer, Gerd; Föll, Dirk; Förster-Waldl, Elisabeth; Gagro, Alenka; Gahr, Manfred; Galal, Nermeen Mouftah; Gambineri, Eleonora; Gamper, Agnes; Gams, Franziska; Ganzow, Astrid; Garcelon, Nicolas; Garcez, Tomaz; GarciaPrat, Marina; Gardiano, Giuliana; Gardulf, Ann; Garibay, Janine; Garwer, Birgit; Gathmann, Benjamin; Gathmann, Jonathan; Gebauer, Corinna; Geberzahn, Linda; Geikowski, Tilman; Geisen, Ulf; Gemander, Christiane; Gennery, Andrew R.; Gerisch, Marie; Gernert, Michael; Gerrer, Katrin; Gerschmann, Stev; Ghosh, Sujal; Giannini, Carolin; Gibbons, Una; Gil Herrera, Juana; Gimenez Sanz, Noemi; Girndt, Matthias; Girrbach, Ramona; Girschick, Hermann; Gkantaras, Antonios; Gkougkourelas, Ioannis; Gnatowski, Susanne; Gnodtke, Elisabeth; Goda, Vera; Goddard, Sarah; Goebel, Daniela; Goffard, Jean-Christophe; Goldacker, Sigune; Gollowitsch, Eva Maria; Gomes, Manuella; Gompels, Mark; Gonzalez Granado, Luis Ignacio; González, Míriam; Gordins, Pavels; Gossens, Lucy; Gowin, Ewelina; Graafen, Lea; Graca, Leo; Gradauskiene-Sitkauskiene, Brigita; Graf, Dagmar; Graf, Norbert; Grange, Elliot; Grashoff, H.Anne; Greil, Johann; Grigoriadou, Sofia; Grimbacher, Bodo; Gronlund, Helen; Groß-Wieltsch, Ute; Gschwend, Cornelia; Guerra, Teresa; Guevara-Hoyer, Kissy; Gueye, Mor Seny; Guibert, Noemie; Guseva, Marina; Guzman, David; Göschl, Lisa; Gößling, Katharina; Gülnur, Birgit; Güngör, Tayfun; Gładysz, Dominika; Haag, Marcel; Haase, Gabriele; Haenicke, Henriette; Haerynck, Filomeen; Hafsa, Ines; Hagin, David; Haliti, Emine; Hallek, Michael; Hammarstroem, Lennart; Hancioglu, Gonca; Handgretinger, Rupert; Hanitsch, Leif G.; Hansen, Susanne; Hariyan, Tetyana; Harrer, Thomas; Hassunah, Pia; Hatzistilianou, Maria; Hauck, Fabian; Hauser, Thomas; Haverkamp, Margje H.; Hayman, Grant; Heath, Paul; Hedrich, Christian; Heeg, Maximilian; Heike, Michael; Heimbrodt, Martin; Heine, Sabine; Heininger, Ulrich; Heinrich, Christian; Heinz, Valerie; Heitger, Andreas; Helbert, Matthew; Helbling, Arthur; Hellige, Antje; Hempel, Julya; Henderson, Karen; Henes, Jörg; Henneke, Philipp; Hennig, Christian; Henrichs, Karin; Herbst, Martin; Hermann, Walter; Hernandez, Manuel; Hernández, Anja; Heropolitanska-Pliszka, Edyta; Herriot, Richard; Herrmann, Friedrich; Herwadkar, Archana; Hess, Christoph; Hess, Ursula; Hesse, Sebastian; Higgins, Sonja; Hilfanova, Anna; Hilpert, Sophie; Hintze, Chantal; Hlaváčková, Eva; Hodl, Isabel; Hodzic, Adna; Hoernes, Miriam; Hoffmann, Christina; Holbro, Andreas; Holtsch, Lisa; Holzer, Ursula; Holzinger, Dirk; Horn, Julia; Horneff, Gerd; Hoyoux, Claire; Hristova, Nataliya; Huemer, Christian; Huissoon, Aarnoud; Hundsdörfer, Patrick; Huppertz, Hans-Iko; Huß, Kristina; Hussain, Sadia; Husson, Julien; Höllinger, Christiane; Hönig, Manfred; Hönscheid, Andrea; Hübel, Kai; Hübner, Angela; Hülsmann, Brigitte; Hüttner-Foehlisch, Tanja; Ijspeert, Hanna; Ikinciogullari, Aydan; Irga, Ninela; İlknur, Kökçü; Jablonka, Alexandra; Jahnz-Rozyk, Karina; Jakob, Marcus; Jakoby, Donate; Jakoby-Gaide, Donate; Jandus, Peter; Jansson, Annette; Jaquet, Melanie; Jardefors, Helene; Jargulinska, Edyta; Jauk, Barbara; Jesenak, Milos; Jilka, Katharina; Jolles, Stephen; Jones, Alison; Jones, Regina; Jonkman-Berk, Birgit; Joyce, Hilary J.; Juliana, Pricillia; Jönsson, Göran; Kabesch, Michael; Kager, Leo; Kahlert, Christian; Kaiser-Labusch, Petra; Kakkas, Ioannis; Kamitz, Dirk; Kanariou, Maria; Kanz, Lothar; Karakoc-Aydiner, Elif; Karanovic, Boris; Kartal-Kaess, Mutlu; Katzenstein, Terese L.; Kayserova, Hana; Kelleher, Peter; Kelly, Dominic; Kentouche, Karim; Kerre, Tessa; Kilic, Sara Sebnem; Kindle, Gerhard; Kirchner, Martina; Kiwit, Simone; Kiykim, Ayca; Klasen, Jessica; Klaudel-Dreszler, Maja; Klein, Ariane; Klein, Christoph; Klein-Franke, Andreas; Kleine, Ilona; Kleinert, Stefan; Klemann, Christian; Klima, Marion; Klocperk, Adam; Kobbe, Robin; Kocacik Uygun, Dilara Fatma; Koch, Melanie; Kochler, Yvonne; Kohistani, Naschla; Kojic, Marina; Kolios, Antonio; Koltan, Sylwia; Kondratenko, Irina; Konoplyannikova, Julia; Kopac, Peter; Kopp, Jana; Korte, Pauline; Kostyuchenko, Larysa; Kracker, Sven; Kramm, Christof; Kramme, Philipp; Krausz, Máté; Kreuz, Wolfhart; Krista, Johanna; Kriván, Gergely; Kropshofer, Gabriele; Krystufkova, Olga; Králícková, Pavlina; Krüger, Renate; Ktistaki, Maria; Kuijpers, Taco W.; Kuis, Wietse; Kullmann, Silke; Kulozik, Andreas; Kumararatne, Dinakantha; Kurenko-Deptuch, Magdalena; KussPaula, Cosima; Käser, Elisabeth; Kölsch, Uwe; Königs, Christoph; Körholz, Dieter; Körholz, Julia; Kötter, Ina; Kühl, Jörn-Sven; Kühn, Alexander; Kümmler, Ria; Kündgen, Andrea; Kütükcüler, Necil; Lafoix-Mignot, Cécile; Lama-Knott, Usha; Lamers, Beate; Lanbeck, Peter; Landais, Paul; Landwehr-Kenzel, Sybille; Langemeyer, Vanessa; Langer, Thorsten; Lankisch, Petra; Lanz, Nadia; Lara, Manrique de; Lara-Villacanas, Eusebia; Laubenthal, Lisa; Laws, Hans-Jürgen; Leahy, Ronan; Lee, Jae-Yun; Lehmann, Andrea; Lehmberg, Kai; Lehner, Patricia; Leibfrit, Hans; Leistner, Leoni; LeMignot, Loic; Lesch, Petra; Leutner, Simon; Liatsis, Manolis; Libai Véghová, Linda; Liebel, Johanna; Liese, Johannes G.; Lilith Staudacher, Olga; Linauskiene, Kotryna; Linde, Richard; Linßner, Martina; Lippert, Conrad Ferdinand; Litzman, Jiri; Llobet, Pilar; Lo, Babacar; Lodin, Tariq; Lokaj, Jindrich; Longhino, David; Longhurst, Hilary; Lopes da Silva, Susana; Lorenz-Uttendorfer, Myriam; Lorenzen, Catharina; Lougaris, Vassilios; Lubatschofski, Annelie; Lucas, Mary; Lunia, Apruva; Lutz-Wiegers, Verena; Löw, Doris; Maaß, Sabine; Maccari, Maria Elena; Mackey, Isobel; Macura-Biegun, Anna; Maerz, Vanessa; Maggina, Paraskevi; Mahay, Balbiro; Mahlaoui, Nizar; Mahrenholz, Hannah; Maier, Sarah; Makhlouf, Mounia; Malfroot, Anne; Malinauskiene, Laura; Mannhardt-Laakmann, Wilma; Manson, Ania; Mantkowski, Felicia; Manzey, Petra; Marasco, Carolina; Marcus-Mandelblit, Nufar; Marg, Wolfgang; Markelj, Gasper; Marodi, Laszlo; Marques, José Goncalo; Marques, Laura; Marschall, Karin; Martinez de la Ossa Saenz-Lopez, Rafael; Martinez-Saguer, Inmaculada; Martire, Baldassarre; Martínez, Natalia; Marzollo, Antonio; Masekela, Refiloe; Masjosthusmann, Katja; Masmas, Tania Nicole; Matamoros, Nuria; Mattern, Jutta; Mau-Asam, Pearl; McDermott, Elizabeth; McGalliard, Rachel; McIntosh, Nichole; Meglic, Karmen Mesko; Meijer, Ruben; Meinhardt, Andrea; Meshaal, Safa; Messaoud, Yasmina; Meyer, Björn; Meyer-Olson, Dirk; Meyts, Isabelle; Micol, Romain; Micoloc, Bozena; Mielke, Gudrun; Mijanovic, Radovan; Milito, Cinzia; Miller, Joanne; Milota, Tomas; Misbah, Siraj; Miskovic, Rada; Mohr, Michael; Mohrmann, Karina; Moin, Mostafa; Molinos, Luis; Morbach, Henner; Moreira, Fernando; Moschese, Viviana; Moser, Olga; Moshous, Despina; Motkowski, Radoslaw; Motwani, Jayashree; Mukhina, Anna; Muller, Eva; Murtra Garrell, Núria; Muschaweck, Moritz; Mutert, Miriam; Mwabe, Brenda; Mödden, Carolin; Möller, Jana; Möller-Nehring, Sarah; Müglich, Carmen; Müller, Christiane; Müller, Gabriele; Müller, Hedi; Müller, Ingo; Müller, Thomas; Müller, Zoe; Müller-Ladner, Ulf; Müller-Stöver, Sarah; Münstermann, Esther; Nademi, Zohreh; Naik, Paru; Nalda, Andrea Martin; Nasrullayeva, Gulnara; Naumann-Bartsch, Nora; Nemitz, Verena; Neth, Olaf; Neubauer, Andreas; Neubert, Jennifer; Neumann, Carla; Niehues, Tim; Niemuth, Mara; Nieters, Alexandra; Nieuwhof, Chris; Nikolic, Branka; Nolkemper, Daniela; Noorani, Sadia; Noorlander, Budde Adya; Notarangelo, Luigi D; Notheis, Gundula; Nowatsh, Sanam Amelie; Näke, Andrea; O'Sullivan, Mary; Obenga, Gaelle; Ocak, Suheyla; Oker, Mehmet; Olbrich, Peter; Olipra, Anna; Omran, Heymut; Oommen, Prasad; Opitz, Linda; Orosova, Jaroslava; Oskarsdottir, Solveig; Pac, Malgorzata; Pachlopnik-Schmid, Jana; Pandolfi, Franco; Papadopoulou-Alataki, Efimia; Papastamatiou, Theodora; Papatriantafillou-Schmieder, Anna; Parmar, Nidhibahen; Parra-Martinez, Alba; Paschenko, Olga; Pasnik, Jarek; Patel, Smita; Pavlík, Martin; Paz Artal, Estela; Pašić, Srdjan; Pearman, Kate; Peeters, Anouk; Pelser, Caroline; Pereira da Silva, Sara Branco; Perez-Becker, Ruy; Perez-Guzman, Marc; Pergent, Martine; Perlhagen, Markus; Peter, Hans-Hartmut; Peters, Nicholas; Petrić, Marin; Pfreundschuh, Michael; Philippet, Pierre; Picard, Capucine; Pichler, Herbert; Pietrucha, Barbara; Pietsch, Daniel; Pietzsch, Leonora; Pignata, Claudio; Piquer Gibert, Monica; Pirolt, Kerstin; Plebani, Alessandro; Pleguezuelo, Daniel E.; Polat, Dilan; Pollok, Katrin; Pommerening, Helena; Popihn, Daniela; Poplonek, Aleksandra; Popp, Marina; Porta, Fulvio; Portegys, Jan; Posfay-Barbe, Klara; Potjewijd, Judith; Poulheim, Sebastian; Prader, Seraina; Prelog, Martina; Prevot, Johan; Price, Arthur; Price, Timothy; Proesmans, Marijke; Provot, Johan; Prämassing-Scherzer, Petra; Pulvirenti, Federica; Quinti, Isabella; Raab, Anna; Raab, Franziska; Rack, Anita; Radcliffe, Ruth; Raffac, Stefan; Rajendran, Nithya; Ramos Oviedo, Eduardo; Randrianomenjanahary, Philippe; Ranohavimparany, Anja; Raptaki, Maria; Rashidzadeh, Roonaka; Rathwallner, Margit; Reda, Shereen; Redouane, Nahida; Regateiro, Frederico S.; Reichenbach, Janine; Reimers, Bianca; Reinhardt, Cornelia; Reinhardt, Dirk; Reinprecht, Anne; Reisli, Ismail; Reiß, Tamara; Renner, Eleonore; Rezaei, Nima; Richter, Alex; Richter, Darko; Rieber, Nikolaus Peter; Rieckehr, Nadja; Riedel, Marion; Riescher, Heidi; Rischewski, Johannes; Ristl, Nicole; Ritterbusch, Henrike; Ritz, Tanja; Rivier, Francois; Robinson, Peter; Rockstroh, Jürgen K.; Roesler, Joachim; Rofiah, Himatur; Rogerson, Elizabeth; Rolfes, Elisabeth; Roller, Beate; Romanyshyn, Yaryna; Rondelli, Roberto; Roosens, Fien; Roth, Johannes; Rothoeft, Tobias; Roubertie, Agathe; Rusch, Stephan; Rutgers, Abraham; Ryan, Paul; Rösen-Wolff, Angela; Rösler, Valentina; Rübsam, Gesa; Sach, Gudrun; Sadeghi, Kambis; Sahrbacher, Ulrike; Saidi, Angelika; Sanal Tezcan, Özden; Sanchez-Ramon, Silvia; Santos, Juan Luis; Sargur, Ravishankar; Savchak, Ihor; Savic, Sinisa; Schaaf, Bernhard; Schaefer, Marzena; Scharbatke, Eva; Schatorje, Ellen; Schauer, Uwe; Scheibenbogen, Carmen; Scheible, Raphael; Scheinecker, Clemens; Schiller, Romana; Schilling, Beatrice; Schilling, Freimut; Schlieben, Steffi; Schmalbach, Thilo; Schmalzing, Marc Thomas; Schmid, Pirmin; Schmidt, Nadine; Schmidt, Reinhold Ernst; Schmitz, Monika; Schneider, Dominik T.; Schneppenheim, Reinhard; Scholtes, Cathy; Schreiber, Stefan; Schrijvers, Rik; Schroll, Andrea; Schruhl, Simone; Schrum, Johanna; Schubert, Ralf; Schuetz, Catharina; Schuh, Sebastian; Schulz, Ansgar; Schulz, Claudia; Schulze, Ilka; Schulze-Koops, Hendrik; Schulze-Sturm, Ulf; Schumacher, Eva-Maria; Schuster, Volker; Schwaneck, Eva; Schwarz, Klaus; Schwarz, Tobias; Schwarze-Zander, Carolynne; Schweigerer, Lothar; Schäfe, Christina; Schölvinck, E.H.; Schönberger, Stefan; Schürmann, Elvira; Schürmann, Gesine; Sediva, Anna; Seebach, Jörg; Seger, Reinhard; Segerer, Florian; Seidel, Markus G.; Selle, Barbara; Seneviratne, Suranjith; Seppänen, Mikko; Shabanaj, Hatidje; Sharapova, Svetlana; Shcherbina, Anna; Shields, Adrian; Shillitoe, Benjamin; Siepelmeyer, Anne; Siepermann, Kathrin; Simon, Anna; Simon, Arne; Simon-Klingenstein, Katja; Simonovic, Marija; Simsen-Baratault, Merlin; Sindram, Elena; Sismanoglou, Nafsika; Skapenko, Alla; Skarke, Maiken; Skomska-Pawliszak, Malgorzata; Slatter, Mary; Smet, Julie; Smith, C. I. Edvard; Sobh, Ali; Sobik, Bettina; Sogkas, Georgios; Sohm, Michael; Solanich, Xavier; Solanich-Moreno, Xavier; Soler Palacín, Pere; Sollinger, Franz; Somech, Raz; Sonnenschein, Anja; Sophie Helnwein, Daniela; Soresina, Annarosa; Sornsakrin, Marijke; Soura, Stavrieta; Spaccarotella, Sabrina; Spadaro, Guiseppe; Sparber-Sauer, Monika; Specker, Christof; Speckmann, Carsten; Speidel, Lisa; Speletas, Matthaios; Stachel, Klaus-Daniel; Stadon, Catherine; Stanislas, Aurélie; Stapornwongkul, Cynthia; Staus, Paulina; Steck, Regina; Steele, Cathal; Steffin, Herbert; Steiner, Urs; Steinmann, Sandra; Stevens, Wim; Stewart, Hannah; Stiefel, Martina; Stieger, Sarah; Stiehler, Sophie; Stimm, Hermann; Stojanov, Silvia; Stojanovic, Maja; Stoll, Matthias; Stoppa-Lyonnet, Dominique; Strapatsas, Tobias; Strauß, Gabriele; Strauss, Timmy; Streiter, Monika; Strik-Albers, Riet; Strotmann, Gaby; Subiza, Jose Luis; Sundin, Mikael; Sutter, Fabienne; SuárezCasado, Héctor; Szaflarska, Anna; Szemkus, Monika; Süß, Birgit; Tamary, Hannah; Tantou, Sofia; Tarzi, Michael D.; Taschner, Helga; Tedgard, Ulf; Teixeira, Carla; ten Berge, RJM; Tenbrock, Klaus; Tesch, Victoria; Tester, Sabine; Tezcan, Ilhan; Thalguter, Sonja; Thalhammer, Julian; Thoma, Katharina; Thomas, Moira; Thomas, Rachel; Thomczyk, Fabian; Thon, Vojtech; Thrasher, Adrian; Tierney, Patricia; Tietsch, Nadine; Tommasini, Alberto; Tony, Hans-Peter; Trachana, Maria; Trapp, Carmen; Trautmann, Victoria; Tricas, Lourdes; Trindade Neves, Maria Conceicao; Trischler, Jordis; Tshilenga, Benite; Tsilifis, Christo; Tönnes, Beate; Ubieto, Hugo; Uelzen, Anett; Uhlmann, Annette; Ullrich, Jan; Ullrich, Kurt; Urbanski, Gerhard; Urschel, Simon; Uszynska, Aleksandra; Vacca, Angelo; Vaganov, N.N.; Vagedes, Daniel; Valicevic, Stefanie; Vallelian, Florence; van Beem, Rachel T; van Damme, Charlotte; van de Ven, Annick; van den Berg, J. Merlijn; van der Flier, Michiel; van Dissel, J.T.; van Hagen, P.M.; van Montfrans, J.M.; van Ogtrop, Geoffrey; van Rens, Jacqui; van Riel, Christel A.M.P.; van Royen-Kerkhof, Annet; van Well, G.Th.J.; Vasiliki, Antari; Velbri, Sirje; Vencken, Jo; Ventura, Alessandro; Vermeulen, François; Vermylen, Christiane; Viemann, Dorothee; Viereck, Anja; Villa, Anna; Vincke, Jeroen; Vinnemeier-Laubenthal, Lisa; Vo Thi, Kim Duy; Voeller, Mirjam; Vollbach, Kristina; Volokha, Alla; Volpi, Stefano; von Bernuth, Horst; von Bismarck, Philipp; Voss, Rebecca; Voß, Sandra; Vural, Yüksel; Wachuga, Heike; Wagner, Norbert; Wagström, Per; Wahle, Matthias; Wahn, Volker; Wapp, Nadine; Warnatz, Klaus; Warneke, Monika; Warris, Adilia; Wasmuth, Jan-Christian; Wasserfallen, Jean-Blaise; Wawer, Angela; Weber, Manfred; Weemaes, Corrie MR; Wege, Lisa; Wehrle, Julius; Weidinger, Stephan; Weiß, Michael; Weißbarth-Riedel, Elisabeth; Welby, Angela; Werner, Antje; Wessel, Sara; Westkemper, Marco; Wicher, Monika; Wickmann, Lutz; Wiegert, Sabine; Wiehe, Monique; Wiehler, Katharina; Wiesböck, Lydia; Wiesik-Szewczyk, Ewa; Williams, Anthony; Williams, Sarah; Winkler, Beate; Winkler, Christel; Winkler, Martina; Winkler, Melanie; Wintergerst, Uwe; Wisgrill, Lukas; Witte, Torsten; Wittkowski, Helmut; Wolf, Barbara; Wolschner, Christina; Wolska-Kusnierz, Beata; Wood, Philip; Workman, Sarita; Worth, Austen; Wortmann, Michaela; Wuillemin, Walter Alfred; Wulffraat, Nico M; Wustrau, Katharina; Wyndham-Thomas, Chloé; Wölke, Sandra; Yasin, Samra; Yegin, Olcay; Yildiran, Alisan; Yilmaz, Denise; Young, Patrick; Yucel, Esra; Zellner, Berit; Zepp, Fred; Zetzsche, Klaus; Zeuner, Rainald; Zečević, Milica; Zielen, Stefan; Zimmermann, Martina; Özsahin, Hülya; Özçeker, Deniz; Ünal, Ekrem; Želimir, Erić.
